# Applying an E-Learning framework to explore learner nurses’ and nurse educators’ perceptions about technology platforms in nursing

**DOI:** 10.1371/journal.pone.0312681

**Published:** 2025-03-18

**Authors:** Masenyani Oupa Mbombi, Mamare Adelaide Bopape, Tshililo Ravele, Tshepo Albert Ntho, Livhuwani Muthelo, Thabo Arthur Phukubye, Tebogo Maria Mothiba

**Affiliations:** Department of Nursing Science, School of Healthcare Sciences, Faculty of Health Sciences, University of Limpopo, Polokwane, Republic of South Africa; University of Southampton, MALAYSIA

## Abstract

**Background:**

Technological platforms provide online support systems for teaching and learning colleges and universities. However, some colleges seem to be not benefiting from these online support systems. The current paper explores the use of technological platforms in nursing education colleges by incorporating the e-learning Context Design Delivery and Outcomes (el-CDDO) framework to demonstrate the benefit of applying the technological platforms. The e-learning Context Design Delivery and Outcomes (el-CDDO) framework has been applied and is beneficial in non-nursing disciplines; however, there has been little scholarly attention paid to the use of this framework to support the delivery of information and communication technology (ICT) platforms in nursing education.

**Aim:**

To adopt the el-CDDO framework in exploring the perceptions of learner nurses and nurse educators about information and communication technology platforms at the Limpopo College of Nursing.

**Method:**

The exploratory-descriptive qualitative research design was adopted to explore the perceptions of learner nurses and nurse educators regarding ICT platforms that support teaching, learning, and assessment in the colleges of nursing within Limpopo Province. Focus group discussions were held with purposively selected learner nurses and nurse educators to collect qualitative data. Thematic qualitative data analysis was used to make sense of collected data according to the five dimensions of the el-CDDO framework.

**Results:**

Six dimensions of the framework highlighted the perceptions of learner nurses and nurse educators about the use of ICT platforms. The perceptions are summarised as the learning context, benefits, enabling and barriers as influencing factors. Benefits include enhanced skills, knowledge, and quick thinking necessary for academic progress and patient care. Enabling factors include the willingness of learner nurses and nurse educators to use ICT platforms. Barriers include insufficient training on ICT platforms, lack of a structured ICT-integrated curriculum, and inadequate ICT skills among lecturers.

**Conclusion:**

The el-CDDO framework proved effective in exploring the perceptions of learner nurses and nurse educators about the use of ICT platforms in nursing education institutions. We recommend using the framework to assess the state of ICT platforms in nursing education institutions.

## 1. Introduction

In today’s rapidly evolving healthcare landscape, incorporating Information and Communication Technology (ICT) platforms is essential to enhance patient care quality, simplify administrative tasks, and facilitate seamless communication between healthcare practitioners [1]. ICT usage is vital in facilitating teaching and learning in nursing education institutions [2]. Thus, there is a rapid adaptation to the digital revolution by changing curricula and redefining the role of nurses as frontline caregivers. ICT encompasses a range of powerful tools, including electronic health records, telehealth systems, mobile apps, and virtual reality simulations. The utilization of these technologies has the potential to completely transform the domains of nursing education, practice, and collaboration [3]. However, the success of these platforms depends on the acceptance and understanding of learner nurses, who represent the future workforce, and nurse educators, who play a critical role in designing nursing curricula. There is rapid growth in the development of ICT facilities that enable learners to search for, build up, analyse and present information, as well as to solve problems [4].

Internationally, The COVID-19 pandemic has led to a transition to online academic programs and increased adoption of the Fourth Industrial Revolution (4IR) in Higher Education Institutions [5]. The 4IR refers to rapid technological advancement in the 21st century [6]. Fourth Industrial Revolution(4IR) focuses on the growing trends towards automation and data exchange in technology, the development of new materials and technologies such as 3D printing, reality, and virtual reality [6]. Furthermore, 4IR encompasses creative organizational campaigns and strategies as well as infrastructure changes to accommodate technology, manufacturing, production, human resources, management techniques, and technologies [7]. Although the current debate is on the 5IR which focuses on technological advancement in small factories [8], including nursing colleges - these developmental trends have not received much attention in the nursing practice and education, hence the purpose of the current paper.

In South-East Asia, there is a lack of focus on incorporating ICT into nursing education [9,10]. In Africa, the integration of ICT into nursing curricula is increasingly used, particularly in Ghana, Uganda, Malawi, Nigeria, Rwanda, and South Africa [2,11–14]. This demonstrates that numerous countries are prepared to embrace progress and innovation in different industries despite coexisting challenges regarding the use of ICT platforms. Hererimana and Mtshali emphasise that learner nurses must be equipped with ICT skills and knowledge relevant to their academic study and professional practice [15]. It is because ICT not only enhances the effectiveness of nurse educators but also offers additional advantages to learners’ creativity. The World Nursing Report (2020) highlighted 10 essential keys to enhance nursing practice and education, emphasizing the significance of science, technology, teamwork, and health equity [16]. For Instance, key four and six emphasise that nursing curricula should equip students to collaborate in interprofessional teams, optimise graduate competencies in health technology, and enhance practice roles by utilising digital health technology. Significantly, in South Africa, various initiatives seek to accelerate the fast implementation of ICT facilities. This includes the National Development Plan 2023 and the Strategic Plan for Nurse Education, Training, and Practice, which call for improving health information systems and using ICT in nursing and midwifery care [17,18]. The health information system is a system that pertains to health data and management, which learner nurses and nurse educators should be aware of [19].

However, efficient implementation of ICT platforms influences the state of awareness by the learner nurses and nurse educators, which requires that human and environmental barriers among others include considering learners’ and educators’ motivation and expectations; and utilising user-friendly technology resources be addressed [20]. To achieve effective implementation of ICT platforms, learner nurses, nurse educators, and nursing institutions must offer adequate training, preparation, and technical support [21]. Notably, the use of ICT platforms in nursing education could offer more advantages among learner nurses and nurse educators during teaching, learning and assessment activities. For instance, it is noted that examining learner nurses' perceptions in nursing colleges is crucial, as the integration of ICT platforms in education caters to diverse learning styles and enhances the learning experience for a broad range of students [22]. There are key elements affecting learner nurses’ perceived satisfaction regarding the use of ICT platforms in three areas; the nurse educator, the course, and the learner [23]. Perceptions of learner nurses and nurse educators about the use of ICT platforms in rural nursing colleges continue to receive less scholarly attention. Therefore, understanding these perceptions can help develop methods for integrating ICT platforms into nursing education and practice, ensuring that the digital tools used align with students’ and teachers’ requirements and preferences. Additionally, the findings of this study add to the continuing discussion about optimising healthcare delivery through technology and moving towards achievements of 5IR, which will ultimately result in better patient outcomes and a more effective healthcare system.

### 1.2. Aim

To adopt the el-CDDO framework in exploring the perceptions of learner nurses and nurse educators about information and communication technology platforms at the Limpopo College of Nursing.

## 2. The e-learning context, design, delivery, and outcomes framework (el-CDDO)

**Fig 1:**
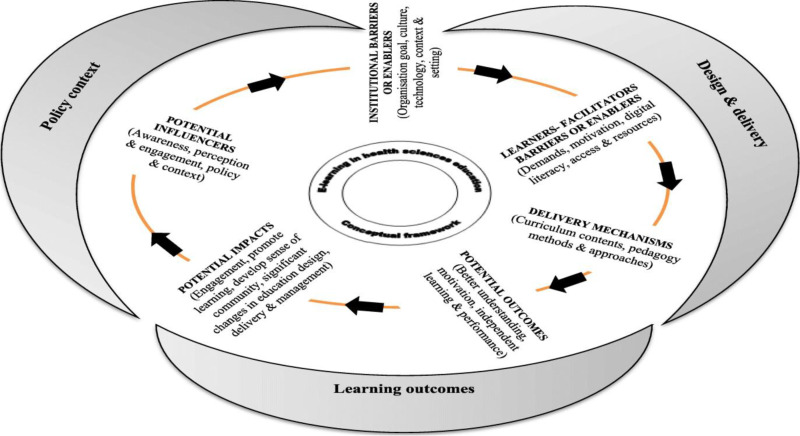
Adopted from Regmi and Jones [20].

The study utilizes the e-learning Context, Design, Delivery, and Outcomes framework (el-CDDO) to present the perceptions of learner nurses and nurse educators on the utilization of ICT platforms for teaching, learning, and assessment. The e-learning Context, Design, Delivery, and Outcomes framework (el-CDDO) is a framework characterised as a stepwise process in a circular flow involving six major components of applying the ICT platforms as depicted in Fig 1 below.

The current paper adopted the six key dimensions of the framework to study the perceptions of learner nurses and nurse educators about the use of ICT platforms in nursing colleges. The dimensions of the framework are described as follows by Regmi and Jones [20]:

### 2.1. Potential influencers

Potential influencers include the awareness, perceptions, and engagement of learner nurses and nurse educators regarding using ICT platforms, alongside the policies and contextual factors at Limpopo College of Nursing. Shahzad, Xu, and Baheer argue that perceived awareness in education significantly enhances the efficacy of ICT-based teaching and learning. This suggests that when learner nurses and educators have a heightened awareness of ICT, they are more likely to utilize these technologies effectively, leading to improved learning outcomes [24].

### 2.2. Institutional barriers or enablers

The institutional barriers or enablers include organisational goals, the culture of the college, context, technology and setting that either inhibit or promote the use of ICT Platforms. Nsouli and Vlachopoulos argue that challenges such as inadequate technology infrastructure and insufficient technological equipment impede the optimal use of technology for teaching and learning in nursing education institutions [25]. To address these issues, Molato and Sehularo emphasize the need for nursing education institutions to implement robust infrastructure supporting ICT platforms for teaching and learning [26]. Additionally, Bester, Smit, De Beer, and Myburgh assert that nursing education institutions must provide ICT infrastructure to support their students effectively [9].

### 2.2. Barriers or enablers related to learners or instructors

The barriers and enablers include the demands, motivation, digital literacy, access and resources related to learners or instructors about the use of ICT platforms. Both learner nurses and nurse educators commonly encounter challenges, such as inadequate preparation for content delivery and restricted access to essential resources needed for integrating ICT effectively into teaching and learning processes [2]. Nursing education institutions’ attitudes and values strongly influence ICT adoption. Borrowing from the Technology Acceptance Model, individuals’ behavioural intention to use technology is significantly shaped by their attitudes and general impressions of the technology [27]. Thus, nursing education’s institutional culture and values play a crucial role in shaping how technology is perceived and integrated into practice.

### 2.3. Delivery mechanisms

Delivery mechanisms encompass curriculum contents, pedagogy, and approaches utilized with ICT platforms. According to Molato and Sehularo, the nursing education curriculum should be revamped to deliver theoretical and clinical components through ICT platforms [26]. Noteworthy, the enhanced delivery mechanism enables more interactive and engaging educational experiences and facilitates the integration of current resources and information into the curriculum.

### 2.4. Potential outcomes

The potential outcomes may encompass enhanced understanding, motivation, independent learning, and performance through the ICT platforms. Alshammari and Fayez Alanazi argue that the potential outcomes of ICT platforms include improved knowledge retention, heightened student engagement, and the provision of flexible learning opportunities [28]. As technology advances, nursing students and educators must utilize its potential to promote better comprehension, motivation, and self-directed learning.

### 2.5. Impact under institutional policy context, instructional design and delivery, and learning outcomes

The section includes engagement, promoting learning, developing a sense of community, and significant changes in the educational design, delivery, and management of ICT platforms. According to Alshammari and Fayez Alanazi, the incorporation of technology has led to significant changes in the educational design, delivery, and management of learning environments, thereby enhancing the overall educational experience [28]. The ICT platforms foster engagement among learner nurses and educators, promote active teaching and learning, and help develop a sense of community among both learners and educators.

## 3. Research methods

A qualitative exploratory-descriptive research design was utilised to explore and describe the perceptions of learner nurses and nurse educators regarding the use of ICT in teaching and learning. The design was relevant to provide insight into learner nurses’ and nurse educators’ perceptions regarding the use of ICT platforms in teaching, learning, and assessment. Adopting the exploratory-descriptive research design enabled the authors to identify enablers and barriers to using ICT platforms in nursing colleges. The descriptive approach assisted the authors in describing the perceptions of learner nurses and nurse educators according to the six domains of the el-CDDO framework. The qualitative research approach relies on carefully presenting daily life as it is experienced by people and stresses the portrayal of human experiences [29].

### 3.1. Study context

The el-CDDO Framework acronym “O” stands for the context where the ICT platforms are implemented. The perceptions presented in this paper are those of learner nurses and nurse educators from Limpopo College of Nursing in South Africa. The Limpopo College of Nursing has five main campuses namely, Thohoyandou, Giyani, Sekhukhune, Sovenga, and Waterberg nursing campus. The study setting for this current paper was at Thohoyandou and Sovenga nursing campuses since the Sekhukhune and Waterberg campuses only offer the first year of the study and the Giyani campus did not give permission.

### 3.2. Population and sampling

The study encompassed all registered 4^th^ year learner nurses and nurse educators at the Thohoyandou and Sovenga campuses of the Limpopo College of Nursing. The group of learner nurses was specifically selected because they are in the last stage of their four-year nursing program, making them well-suited to share their thoughts on the utilization of ICT during their studies. The fourth-level learner nurses had enough opportunities to use ICT platforms than those in 1^st^, 2^nd^ and 3^rd^ levels of studying, hence the authors only focus on the final level learner nurses. Meanwhile, nurse educators play a role in facilitating teaching and learning, which includes applying ICT platforms in teaching and assessment, and motivating the learner nurse’s innovativeness during their learning was the main reason that necessitated the selection of these groups to the study. A total of forty-seven learner nurses and sixteen nurse educators took part in the study.

### 3.3. Data collection

Data were collected from two different groups of people; the learner nurses and nurse educators who were purposively recruited for participation in the study from 18 August 2019 until 30th August 2019. Authors adopted non-probability, purposive sampling to select only learner nurses of the 4^th^ level, considering that they are in the final year of studies that exposed them to various learning contents that require the use of ICT platforms and that they can be readily available for interviews as a group. The purpose of including the nurse educators was primarily because they are the sole providers of teaching and learning, and we aimed to obtain their perceptions regarding the use of ICT platforms when teaching. Since the study was purely qualitative, data was collected using focus group interviews from 1^st^ of September to 18 December 2019. A Focus group interview is a qualitative data collection technique that consists of six to ten individuals sharing common characteristics of interest to the study [30]. The focus group benefited the authors by allowing the two groups of participants to share their perceptions, generate a debate and obtain meaning regarding the use of ICT platforms in three selected colleges. The focus group interviews were conducted by experienced authors with good research and communication skills in qualitative research and conducting focus group interviews. The authors used an interview guide as a tool to direct the interviews, focusing on an exploration of the perceptions of learner nurses and nurse educators regarding ICT facilities in teaching and learning. Each focus group consisted of 5 to 10 leaner nurses and 6 to 8 nurse educators. The researcher had three focus group interviews with learner nurses and one focus group interview with nurse educators on the Thohoyandou campus: three focus group interviews with learner nurses and one focus group interview with nurse educators on the Sovenga campus. The interviews were held on the respective campuses in a quiet environment, free from interruptions. The duration of focus group interviews was between 35 minutes and 1 hour. An audio recorder was utilised, and field notes were collected. The primary author asked the group one central question: “**Can you please tell me about your perceptions regarding the use of information and communication technology facilities in teaching and learning?”** Subsequent questions were guided by the responses to the initial question. Probing skills, such as minimal verbal responding, clarifying, reflecting, focusing, paraphrasing and validation, were used to acquire more information [30]. The interview process was explained beforehand, and only started when participants had given voluntary consent to their participation.

### 3.4. Data analysis

The thematic content analysis approach was utilized to identify, examine, and report patterns/themes according to six steps within the qualitative data gathered [31]. Step 1 (Familiarisation of data) -All audio-recorded interviews were captured verbatim in a Microsoft Word document. Four primary authors carefully read through the transcriptions of each interview to get a sense of the research. Step 2 (Generating initial codes) - Interview transcripts and field notes were analysed to generate a list of similar topics that were clustered together and formed into columns. Step 3 (Searching for themes) -Related topics were grouped into categories. Step 4 (Reviewing themes) - The main author collected the data from each category and then conducted a preliminary analysis to identify the themes and sub-themes. Step 5 (Defining and naming the themes) - The study results were examined, and the final topic and sub-themes were defined and summarized after the co-authors listened to the recorded interviews and transcribed data. Step 6 (Producing the report) - The detected themes and sub-themes were less inconsistent, which caused other themes to merge into one theme. The topics and sub-themes were confirmed by the remaining four authors, and a decision was made by all of them. The emerged themes and sub-themes about the perceptions of participants on the use of ICT platforms were categories according to the six domains of the el-CDDO framework.

### 3.5. Measures to ensure trustworthiness

Four criteria for trustworthiness, as outlined by Botma, Greeff, Makhado and Mulaudzi are used to establish the trustworthiness of the study [32]. Credibility was ensured by prolonged engagement in the study, the use of audio recordings to record the data, and the taking of field notes during the unstructured interviews. Independent coding of the results was done by the second author to enhance dependability. To ensure confirmability the transcripts and the recorded tape were sent to an independent coder to conduct an inquiry audit on the data, and the meaning attached to it. The authors used a purposive sampling method to enhance transferability when selecting learner nurses to participate in the study.

### 3.6. Ethical approval

The study was ethically approved by the Turfloop Research Ethics Committee (TREC/135/2019:PG). Permission to collect data at the Thohoyandou and Sovenga campuses of the Limpopo College of Nursing was obtained from the acting vice-principals on both the Thohoyandou and Sovenga campuses. The participants were informed about the aim and objectives of the study and that participation in the study was voluntary. All the participants who agreed to participate in the study were asked to sign a voluntary informed consent. This study adhered to this ethical consideration by ensuring that information provided by respondents and/or participants was safely stored and was never shared with other people. To safeguard the privacy of the participants/respondents, they were interviewed in a private environment, away from passers-by or intruders [30].

## 4. Results and Discussion

### 4.1. Demographic data of participants

Tables 1 and 2 below present demographic data of learner nurses and nurse educators who participated in the study. To cover the scope of utilization of technological platforms – the authors saw it significant to collect data from the two groups; nurse educators and learner nurses within the nursing colleges which can enhance the trustworthiness of the data and demonstrate areas of convergence regarding perceptions on the use of ICT platforms for teaching, learning and assessment in nursing colleges.

**Table 1:** Demographics of the learner nurses who participated in the study.

Variables of learner nurses	Type of variable	Frequency	%
Gender	Male	10	21
	Female	37	79
Age	21-25 years	42	89
	26-30 years	5	11
Highest qualification	Matric	43	91
	Diploma	4	9
Total	**Learner nurses**	**47**	**100**

**Table 2:** Demographics of the nurse educators who participated in the study.

Gender	Male	1	6
	Female	15	94
Age group	30-39 years	9	56
	40-49 years	5	31
	50-59 years	2	13
Years of teaching practice	0-5 years	9	56
	6-10 years	5	31
	11-20 years	2	13
Highest qualification	Diploma in nursing education	2	13
	Bachelor of Nursing	1	6
	Masters in Nursing	12	75
	PhD	1	6
Total Participants	**Nurse educators**	**16**	**100%**

Tables 1 and 2 above illustrates that a total of 63 participants from a nursing education institution participated in the study. For instance, Tables 1 and 2 shows a total of 47 learner nurses and 16 nurse educators were recruited and interviewed in the study. Most of the participants` leaner nurses were females, constituting 79% (37) of the total number of participants, while males constituted 21% (10) of the participants. On the other hand, nurse educators` participants were dominantly females, 94% (15) with the remaining 6% (1) being males. These differences just prove that nursing profession in South African context is still dominated by the female gender as they dominated in both the group of learner nurses and nurse educators.

Most learner nurses were between 21 and 25 years of age, while only 5 learner nurses were between 26 and 30 years of age. Nine (56%) nurse educators were between 30 and 39 years of age, 5 (31%) were between 40 and 49 years of age, and only 2 (13%) were between 50 and 59 years of age. In terms of age differences, both the age group in learner nurses and nurse educators were still young 21-25 and 30-39 respectively. One would assume that this is the age group that is ought not to struggle with the use of ICT.

The qualifications of nurse educators were mostly a master’s qualification 75% (10) with only 6% (1) holding a PhD degree. This means that the nursing education institutions where the study was conducted were adequately qualified, with the staff possessing advanced knowledge, specialized skills, and personal qualities that set them apart in the professional world and allowed them to deal with a shift into the 4^th^ industrial revolution. Most of the nurse educators were novice educators 56% (9) with less than five years of working experience and once again, a group that is fresh from post-graduate studies where the use of technology is the order of the day for research activities.

### 4.2. The el-CDDO framework and its application to explore perceptions of learner nurses and nurse educators

This framework can also be called the ‘e-learning CDDO (Context, Design, Delivery, And Outcomes) configuration framework’ in Health System Education. The framework is used to describe the perceptions of nurse educators and learner nurses. The el-CDDO framework is characterized by five major components:

#### 4.2.1. Potential influencers.

Learner nurses indicated that ICT facilities equip them with advanced skills, knowledge, and independence leading to self-directed learning. Furthermore, learner nurses described ICT as a facility providing diverse opportunities for learning which makes it easy for them to access and share information at the various levels of learning.

*• Diverse existing opportunities to implement the ICT platforms mentioned*

The findings of the current study revealed existing and diverse opportunities for the implementation of ICT platforms, which can assist in the implementation of ICT. The authors discovered that the college has an association with the University of Limpopo which is well-equipped with ICT facilities that could play a significant role in teaching, learning, and assessment. Learner nurses further reported that there are computers on their campuses that are not being utilized, which could be an opportunity for the utilization of ICT platforms. This finding was supported by the following quotes - see S1 for sample data from the respondents:

… *We are in association with the University of Limpopo which in my view I think is well equipped in terms of ICT… (Participant 3).*

*So we are having one of the best universities I mean the internet there…one of the best libraries…We do have a laboratory there and there are computers which are still new…(Participant 6).*

*…there are many computers [at the campus]…but I don’t know when they are being utilized…(Participant 2).*

Willemse and Bozalek mentioned that incorporating ICT devices into nursing education programs provides learner nurses with a rich resource of up-to-date reference material to use in both classroom and clinical settings [33]. Wirihana, Craft, Christensen and Bakon outlined the notion that the use of video learning has the potential to enhance clinical and classroom experience, in that nurse educators can disseminate information to, and reach, a large number of learners through podcasting [34]. Willemse stated that flexible learning affords learners the choice of where, when and how they can manage their learning [35]. Flexible learning gives learners access to an ‘e-educator’ whenever, wherever and however guidance and clarification are needed.

*• Educators are willing to implement and use ICT platforms in teaching, learning, and assessment*

The study findings indicate that educators are willing to implement and use ICT platforms in teaching, learning, and assessment. The authors discovered that nurse educators utilize their own means to enhance communication with learner nurses in teaching, learning, and assessment. Nurse educators reported that they use their own Internet data to provide visuals to learner nurses for their learning. This was reflected in these statements (see S1 for sample data from the respondents:):

*…We do try as lecturers sometimes but we are using our means like we have our WIFI. I even went to an incredible connection to buy an antivirus called Capeskin (Participant 6).*

*The other thing is when we want to communicate with our students we have to use our cell phones because we don’t have this information technology here”(Participant 4).*

*I use my own money and internet for the class when I want to teach the students using visual learning…and I’m not being compensated (Participant 9).*

In support of the current study findings, Goldschmidt and Colleagues stated that nurse educators develop learning activities using videos that evaluate knowledge; leading learners to question their values or behaviours and to focus on the performance of a particular psychomotor skill [36]. In addition, Kala et al. stated that nurse educators must design educational experiences that address knowledge and clinical decision-making skills [37]. Therefore, Reyes, Reading, Doyle and Gregory, suggested that educators and academic developers may use blended learning methods to enhance the acquisition of both cognitive knowledge and practical skills in health disciplines [38].

#### 4.2.2. Institutional barriers or enablers.

The authors describe the challenges experienced by learner nurses related to the use of ICT facilities at the LCN. Learner nurses indicated that the lack of skills of nurse educators in utilizing ICT facilities makes it difficult to implement ICT in teaching and learning on LCN campuses. Additionally, learner nurses reported the lack of a structured curriculum that embraces ICT platforms, which leads to a lack of implementation of ICT in teaching and learning at LCN.

*• Lack of an ICT department to facilitate the usage, and availability of proper ICT infrastructure and platforms*

Both participants indicated that a lack of an ICT department to facilitate the usage, and availability of proper ICT infrastructure and platforms prevents the implementation of ICT in teaching and learning. Participants indicated that there is no ICT department on the campuses of the LCN to facilitate ICT use and maintain the equipment. The participants further reported that no training or in-service support was provided on how to utilize ICT facilities. Both sets of participants indicated that a lack of an ICT department to facilitate the use and maintenance of the ICT platforms prevents the implementation of ICT in teaching and learning. Participants indicated that there is no ICT department on the campuses of the LCN to facilitate ICT use and maintain the equipment. The participants further reported that no training or in-service support was provided on how to utilize ICT facilities. The authors discovered that learner nurses do not have a strong desire to use ICT facilities because of the lack of proper ICT facilities in college libraries. Furthermore, learner nurses reported that they receive their examination results posted on notice boards, which are only accessible on the campuses. The findings are supported by the following quotes (see S1 Sample data from the respondents):

*We [educators] don’t even have a technician on campus so if you are having problems with computers you have to consult fellow lecturers or maybe sometimes students to come and assist you….there is no such department here we say this is our IT department, so I believe if we have such department we were not going to be lacking on [ICT (Participant 3).*

*…a technician must come to the offices maybe once a month to come and fix whatever is broken and then the other thing, I think we should have the workshops where they teach us about the new developments about information technology…antivirus needs to be updated so if it’s not updated you can have…the virus in the PC we need ICT (Participant 9 and 4).*

*…having a person who is dealing with ICT services for both lecturers and the students to have WIFI even in the classroom then it will improve our lesson plans on our facilitation in classrooms (Participant 6).*

The lack of an ICT department to facilitate the usage, and availability of proper ICT infrastructure and platforms was identified as a major challenge that hinders the effective use of ICT platforms in teaching and learning by both educators and learner nurses. Similarly, Kala et al. found that a lack of proper ICT facilities will hinder students from enjoying the benefits of ICT, which include increased learner motivation and satisfaction, engaging in active teaching-learning methods and resulting in enhanced cognitive recall [37]. Moniz, Pereira, and Marques supported these findings by stating that ICT stimulates learners’ interest, reflection and participation in a relaxed manner, while allowing the use of existing knowledge; afford learners a chance to express themselves and their points of view on their own [39]. Therefore, Chipps, Pimmer, Brysiewicz, Linxen, Ndebele and Gröhbiel recommend that educational institutions should support learners to use of technology to perform academic activities, especially concerning the development of mobile literacy skills [40]. Similarly, Ricks et al. stated that continuous technical support from information technology staff is required during the implementation of ICT so that users could gain access to information at point-of-care because learners were not competent in using ICT [41]. Bozdogan and Özen supported this finding by stating that educator-training technology components are important factors in ICT self-efficacy level [42]. Courses offered to educators must cover areas of ICT-based equipment development and learners’ hands-on teaching activities while maintaining current updates. Livingstone further stated that ICT tools are enablers in the learning dynamic, in terms of content appropriation when developing learners and creating interesting platforms that promote interaction and information sharing [43]. Therefore, Willemse suggested that mobile devices should be used to enhance teaching and learning since they can become preceptors, shadowing educators; bringing them closer to the students in the clinical facilities [35].

*• A lack and non-maintenance of those existing ICT resources at LCN are perceived as problematic for the implementation of fruitful teaching, learning, and assessment aspects*

The current study findings provided information on the lack of ICT resources at the LCN, which is problematic for the implementation of fruitful teaching, learning and assessment. Also, the participant reported that there are existing ICT facilities that are non-functional because of a lack of maintenance, thus inhibiting the continuous professional development of lecturers. Furthermore, nurse educators reported that they do not have any software to assist them in scheduling marks for learner nurses, which they have to do manually. The findings are supported by the quotes below (extracted from see S1 for sample data);

*I believe if we need to use this information communication technology we need to have facilities like WIFI in the institution which is not there (Participant 6).*

*We do not have antivirus so some of us…we’ve installed our antivirus on the campus computers (Participant 9).*

*…we are still using a pen to write students’ final marks. We do not have software or whatever that can help us, even ordinary excel is not being used in our college (Participant 7)… if you want to use videos to show students videos are difficult because we don’t have resources (Participant 5).*

In support of current study findings, Clifton and Mann stated that classrooms equipped with Internet and projectors provide educators with quick access to YouTube to utilize videos to meet their classroom objectives [44]. YouTube offers educators the opportunity to alternate between traditional teaching methods of teaching and provide visual stimulation that captures and maintains the attention of students in the classroom. Therefore, Button et al. recommended that nursing education leaders should provide access to technology and adequate computer resources for educators to integrate nursing informatics into their professional activities [45].

#### 4.2.3. learners or instructors – barriers or enablers.

Learner nurses indicated that there is a lack of training for both learners and nurse educators on the use of ICT in teaching and learning, which makes it impossible to implement the use of ICT facilities for teaching and learning on their campuses. Furthermore, learner nurses described the poor ICT infrastructure, which makes it even more impossible to implement ICT in teaching and learning at the LCN.

*• Existing ICT platforms not used by learners and educators that is perceived as wasting of resources*

Both sets of participants of the study indicated that learners and educators, which they viewed as wasting of resources, do not use existing ICT platforms. It was reported that learner nurses do not use computer laboratories because they are not given academic activities that require them to make use of such facilities, except for research. Additionally, the learner nurses indicated that only a few desktops are available to them in the library and the library is closed while they are attending classes. These findings are supported by the quotes below (extracted from see S1 for sample data);

*We [learners] have a computer lab but the way our course is, we do not have [academic activities], we do not see it of value that we can go to the computer lab (Participant 3).*

*…we [learners] came here in 2016 … we found those computers there and we don’t even know if they are working or not… (Participant 2).*

*…Mostly the library closes at 16:30 and we [learners] are in class between 7:00 and 16:30 then you should be in class..(Participant 12).*

Existing inadequate ICT infrastructure and lack of training for both students and nurse educators on using ICT in teaching and learning, make it impractical to implement ICT facilities on their campuses.. ICT platforms not used by learners and educators that is perceived as wasting of resources. In agreement with the findings, Willemse and Bozalek stated that incorporating personal digital assistance (mobile devices) into nursing education programs provides learners with a rich resource of up-to-date reference material to use in both classroom and clinical settings [33]. Wirihana et al. supported the finding by stating that the use of video learning enhances clinical and classroom experience, educators can disseminate information and reach a large number of learners through podcasting and vodcasting audio and video files [34]. These disseminated files can be transferred to computers and mobile devices. Dewah and Mutula suggested that, since most learners own smart cell phones, educators could encourage learners to use their cell phones to engage in educational tasks [46].

*• Lack of skills by lecturers to facilitate utilization of ICT platforms mentioned as problematic in increasing its uptake outlined*

Learner nurses reported that the lack of skills by nurse educators to facilitate the use of ICT facilities is problematic for teaching and learning. Therefore, nurse educators are challenged with operating media devices, such as data projectors, while some have difficulty using email and WhatsApp as a means of communication. This was reflected in the following statements (extracted from see S1 for sample data):

*Even the lecturers don’t even have skills on how to operate these new technology things… (Participant 1).*

*… lecturers…use data projectors [is not good] … When the lecturers try to make them work you find it’s just a blank blue colour on the white sheet (Participant 11).*

*…lecturers do not even know how to use c[send] email. Some of them do not even know how to use WhatsApp…if they want to pass on a message they call the class rep… (Participant 14).*

Hebda and Calderone stated that training for both learner nurses and nurse educators should not be regarded as unimportant, since it is crucial for the success of ICT implementation [47]. Palomino supported this finding by stating that educators are not advised on how to search for information, or how to select and assess ICT tools to develop the teaching-learning process [48]. Garrison, discovered that nursing education leaders do not provide access to technology and adequate computer resources to encourage the integration of ICT into nursing activities [49].

#### 4.2.4. Delivery mechanisms.

*• Lack of training of all LCN stakeholders on the use of ICT platforms is problematic*

Learner nurses indicated a lack of training of all LCN stakeholders on the use of ICT platforms as problematic. The authors discovered that the campuses do not have people who are trained on how to utilize ICT facilities. Furthermore, learner nurses indicated that educators are old age and are challenged by the use of technology; with only one person available to assist all learners with ICT-related issues in the library. The findings are supported by the quotes from the participants below (extracted from see S1 for sample data);

*… we lack people who are well equipped [with ICT knowledge and skills] who can help us on how to use this ICT (Participant 3).*

*…we [Learners] don’t know how to use a computer, you get to the library you want to learn how to use a computer you get there only to find one person on assisting (Participant 22).*

*…we [learners] are having lecturers who are old fashioned; their skills are not well [aligned] to the new technology things, it’s very hard for them to fit in the new technology…(Participant 5).*

Fiedler, Giddens and North identified a lack of technical skills associated with ICT as a barrier that institutions must overcome [50]. To adopt new technologies, educators need training on how to use technology. In agreement with Giddens and North, Ricks, Benjamin, and Williams reported that, due to a lack of training in ICT, learner nurses are unable to demonstrate computer skills, such as how to type using a keypad; and how to download and save information [41,50].

• *Lack of structured curriculum that embraces ICT platforms blamed for lack of implementation pointed out*

The findings of this study indicate that the lack of a structured curriculum that embraces ICT platforms is responsible for the lack of implementation of ICT in teaching and learning. The authors discovered that the curriculum offered by the LCN does not cater to ICT utilization in teaching and learning. In addition, learner nurses reported that their modules do not involve technology in any way. These findings are supported by the quotes below (extracted from see S1 for sample data)

*There’s no single module that involves a computer, if technology was taken serious, a module that teaches computers would be available (Participant 2).*

*If we [learners] can have once in a year or week where they will teach people who are computer literate on how to operate computers and stuff (Participant 22).*

*…they can choose at which level they can teach you the computer and focus only on the computer literate...(Participant 20).*

The absence of a well-defined curriculum that integrates ICT platforms is accountable for the limited adoption of ICT in teaching and learning. The authors found that the curriculum provided by the LCN does not support the use of ICT for teaching and learning purposes. Additionally, learner nurses indicated that their study modules do not incorporate technology in any form. Dickens explains that the integration of ICT into the nursing curriculum allows educators to facilitate ICT use in the classroom and clinical settings [51]. Button et al. supported the findings by stating that e-learning has been introduced to the nursing curriculum in Western countries, including into the curriculum for advanced learner nurses and nurse educators supported by ICT [45]. Bembridge, Levette-jones and Jeong recommended that failure to include technology-based modules in the curriculum be rectified so that the curriculum includes competencies in the use of ICT in a teaching and learning environment [52].

• *Correlation of theory into practice related to the use of ICT platforms in teaching, learning, and assessment outlined*

Both participants of the study described the correlation of theory into practice related to the use of ICT platforms in teaching, learning, and assessment. The authors discovered that the lack of ICT implementation in teaching and learning has an impact on the integration of theory into practice. Participants reported that due to a lack of resources, simulation is mostly used during clinical teaching. Participants further reported that ICT facilities award them an opportunity to watch the procedures online repeatedly. The finding is supported by the following quotes (extracted from see S1 for sample data):

*…we are teaching learners how to cram and go but there is no integration of the real practice with the theory that they are learning because of some of the conditions or procedures you might not find in the wards but online (Participant 5).*

*…sometimes in the practical places you are alone and the lecturers and the sisters are not there if you are using the technology it might help you to find the information you are looking for (Participant 7).*

*…with technology, you can watch a procedure over and over again…go to YouTube watch whatever the lecturer showed you in the class (Participant 9).*

The study revealed that limited adoption of ICT in teaching and learning affects the integration of theory into practice. Participants mentioned that, because of resource constraints, simulation is predominantly used during clinical teaching. They also noted that access to ICT facilities allows them to review procedures online repeatedly. In support of the current findings, Willemse and Bozalek stated that introducing learning using mobile devices (m-learning) to undergraduate nursing programs assists learners in bridging the gap between theory and clinical practice [33]. These authors further supported this finding by stating that email and WhatsApp are appropriate tools to be used for the integration of theory and clinical practice [33]. By using these tools, educators can attach short videos or slides via email and provide learners with pictures or case-based scenarios via a WhatsApp group to facilitate self-directed learning. Furthermore, Nyangeni, Du Rand and Van Rooyen stated that the use of social media can improve clinical practice by providing learners and educators with guidance from specialists throughout the world, since they can share pictures or communicate through messages [53]. This will ensure that they receive the best guidance on the provision of better patient care. Willemse concluded that social media applications provide learners with a support structure to enhance the integration of theory and clinical practice since these applications maintain academic support, information sharing and collaborative practice [35].

#### 3.2.5. Potential outcomes.

• *Use of ICT facilities in nursing science is perceived as equipping nurses with advanced skills, knowledge, and information for patient’ care*

The findings of the study indicated that the use of ICT facilities in nursing science is viewed as equipping nurses with advanced skills, knowledge, and information for patients’ care. The authors discovered that learner nurses find it easy to obtain information and to learn new skills when using the Internet, which assists them to access procedures on YouTube, providing on-the-spot in cases when they need to be remaindered of a procedure, using their cell phones. Learner nurses also reported that they keep abreast of what is happening worldwide. This was reflected in these statements (extracted from see S1 for sample data);

*It is easy to learn skills on the internet you [learner] can just quickly go to your YouTube they always have skills there on how to do certain skills…(Participant 15).*

*…if maybe you [learner] don’t know certain procedures you can just go through your phone and see how they do the procedure…(Participant 10).*

*…We [learners] become more and more knowledgeable…we get updated on what’s happening far worldwide, so with that you grow and you learn new things (Participant 9).*

In support of the current study findings, Ricks et al., explained that the introduction of ICT into healthcare facilities allows learners and educators to access recent clinical knowledge needed at the point of care of patients [41]. Bloomfield and Jones supported this finding by stating that ICT is valuable for developing clinical skills, and video clips are perceived as the most useful feature for learning [54]. Palomino further stated that smartphones allow learner nurses to access clinical information, allowing them to have more references at hand and to access prescription rules, drug references, and interactions while consulting patients at point-of-care [48].

• *ICT facilities are perceived as useful for easy, quick thinking and provision of timeous implementation of world-class care to patients.*

The findings of the study indicate that ICT is useful for easy and quick thinking and for the provision of timely implementation of excellent care to patients. Learner nurses reported that ICT facilities provide them with quick solutions in times of need. Learner nurses further indicated that they can keep abreast of their profession when using ICT facilities by being able to health conditions in other countries outside South Africa. The finding is supported by the quotes below (extracted from see S1 for sample data);

*Even when I’m in the clinical area they ask you [learner] a question you don’t understand you can quickly go to your phone on the internet and search for it (Participant 22).*

*With technology, I [learner] get new information very fast and the things that are updated …(Participant 23).*

*We also get to know other health conditions that are affecting other countries…not only focusing on South Africa…(Participant 9).*

In support of the findings of the current study, Palomino argued that ICT equipment is an enabler in the learning environment in terms of promoting active participation; while facilitating individual, cooperative and interactive work in class and the clinical environment; which, in turn, enables learner-educator communication [48]. Chipps et al., discovered that smartphones can be used for educational purposes, to provide quick access to educational resources and guidelines during class and clinical activities, or to support nurses at the bedside [40]. Bloomfield and Jones found that direct links to articles provide learners with evidence-based information and videos demonstrating clinical skills [54]. Willemse strongly motivated the use of WhatsApp group discussions as a means to assist in understanding the application of theory during clinical practice, serving as a supporting platform for the integration of theory and practice [35].

• *ICT facilities facilitate self-directed learning and independence, which makes students’ learning easy*

The findings of the current study indicate that ICT facilities facilitate self-directed learning and independence, which leads to learning becoming easy for students. Learner nurses reported that, with ICT facilities, they can go to podcasts to reinforce what was learned and, therefore, their independent learning is facilitated. The finding is supported by the following quotes (extracted from see S1 for sample data);

*…if you have time you go through your internet or maybe podcast on that certain topic, it will help you to understand (Participant 8).*

*Like I can teach myself how to email, and how to use those things like how to search for certain things. If we doing school work I can google things, assignments and all…(Participant 17).*

*So I think we learn differently and if you check these days we are more used to tablets, we are more used to phones we are used to using technology and it makes it convenient…(Participant 15).*

In support of current study findings, Cook, Levinson and Garside stated that e-learning increases learner’s control over the content, place and time of learning, learners can gain skills and knowledge faster than when using traditional methods [55]. The work by Button et al. supports this finding by stating that online learning platforms provide flexibility and self-paced learning for learners enjoy the online learning environment because they get to know each other and to assist each other [45]. Wirihana et al. support the finding by stating that video learning does not only promote lecture dissemination, it also meets the specific needs of the learner by promoting learner accountability, autonomy, motivation and satisfaction [34]. Learner nurses can attend classes, participate in discussions, and complete assignments through a website [56]. In this section, the authors analysed the perceptions of learner nurses regarding ICT facilities in teaching and learning. The findings revealed that the major challenges identified by the learner nurses were the unavailability of ICT facilities, the lack of skills of educators, the unavailability of connectivity, and the lack of training for nurse educators.

• *ICT platforms are perceived as relevant in aspects of teaching, learning, and assessment though not implanted causing challenges on different levels*

The findings of the study indicate that nurse educators perceived ICT platforms as relevant to aspects of teaching, learning, and assessment though not implemented. Furthermore, nurse educators reported that they have to physically deliver assignments when learners are in clinical practice because of a lack of ICT. These findings are supported by the quotes below (extracted from see S1 for sample data);

“*ICT on its own it’s quite relevant to the institution, particularly looking at the era that we are in, but unfortunately in our institution, it’s not practical (Participant 8).*

“*if there was the technology we could post videos showing the students how suctioning is done (Participant 9).*

*I have read that technology enhances education, and promotes critical thinking amongst students (Participant 5).*

Raman explained that the use of mobile technology improved nursing students’ learning and performance in the clinical setting by providing them with easily accessible and current evidence-based facts [57]. Supporting this finding, Holland, et al., stated that ICT strategies, such as video use, assist in the development of the psychomotor clinical skills that are critical for patient care, videos provide a visual demonstration of clinical skills in a simulated close-to-real setting [58]. Goldschmidt and Colleagues further stated that technology use in nursing education is widely encouraged, as nursing education has adapted to include the use of simulation, gaming, virtual reality, and interactive case studies that can teach acute care in a safe learning environment [36].

• *Educators expressed concern about producing learners who are not familiar with ICT*

The findings of the current study described concerns raised by nurse educators on training students who are not familiar with ICT. The authors discovered that nurse educators are concerned about not being able to cater to all students because some students are unable to use ICT in teaching and learning. The nurse educators further reported that they feel that their lessons are not interesting since there are no visuals to stimulate the learner nurses. The findings are supported by the following quotes (extracted from see S1 for sample data);

*…we [are just producing learners who just have a theory … they have never seen it in the ward, some they have never seen it in the videos …students assumed from level 1 up level 4 (Participant 9).*

*I feel that the lessons are boring because there’s not much to view…our lessons are not interesting because of a lack of resources (Participant 5).*

Serhan stated that, because of technology, there is a shift from direct face-to-face contact between educators and learners to increasingly virtual contact [59]. In support of these findings, Johansson, Petersson et al., stated that new technology may enable new services; meaning that nurses will be able to conduct virtual visits over the internet and discuss a patient with other health and social care professionals through a secure connection [60]. Furthermore, Button et al. argued that nursing curricula must aim to prepare nursing graduates who are ‘knowledge workers’ who are able to manage information and sophisticated technology, on the one hand, and make complicated clinical judgments, on the other [45]. Therefore, Bembridge, et al., recommended that nurse educators should look for ways to enhance the learning environment to fit the expectations of learners; and to ensure the transferability of ICT skills learned at college into the healthcare workplace [52].

## 5. Conclusion

The paper aimed to adopt the el-CDDO framework in exploring the perceptions of learner nurses and nurse educators about information and communication technology platforms at the Limpopo College of Nursing. The el-CDDO provides a structured and comprehensive approach to developing e-learning programs that are learner-centered, contextually relevant, well-designed, effectively delivered, and outcome-focused. The study indicated that the use of ICT facilities in teaching and learning is a concern for both learner nurses and nurse educators. The most frequently used ICT facilities by learner nurses and nurse educators for teaching and learning at LCN are projectors, laptops, and mobile technologies (smartphones). Emails are not being used by educators for teaching and learning purposes. Due to a lack of ICT facilities on the campuses, technologies like video transmission and video conferencing are not used. Learner nurses are challenged by the unavailability of ICT facilities and a lack of training on how to use ICT for learning purposes. Although educators are willing to implement ICT in teaching and learning, they are hindered by several factors, including inadequate ICT facilities; a lack of training for the newly employed and older educators; and the unavailability of network connectivity. This study highlights how learners without access to ICT facilities are deprived of the opportunity to use current, evidence-based information and to interact with one another. Therefore, the Limpopo College of Nursing should design measures to ensure that campuses have ICT facilities installed for facilitation of teaching and learning.

## 6. Recommendations of the study

The following recommendations are based on the themes that emerged during the interviews with learner nurses and nurse educators. Although learner nurses and nurse educators realize the importance of ICT, it is not currently rooted in nursing education at the LCN. The study indicates that it is important that the college management work towards providing an environment that supports the use of ICT for teaching and learning. The following headings discuss the recommendations made from the findings and suggestions received from both learner nurses and nurse educators aimed at addressing the challenges encountered by the use of ICT in teaching and learning at the LCN.

- The college management should initiate the provision of ICT facilities that can serve all campuses. The LCN should benchmark how the ICT platforms are managed in other higher education institutions.
- Provision of ICT facilities, such as the Internet and laptops for nurse educators, and desktops for learner nurses, as part of college equipment, should be a basic requirement.
- All classrooms for teaching and learning, as well as the library, should be equipped with the requisite ICT facilities for easy access and in support of teaching and learning activities.
- To ensure continued and reliable access to the Internet, and a mechanism needs to be found to ensure the serviceability of the computers and softwares within the college.
- ICT experts and technicians should be employed and be easily accessible to learner nurses and nurse educators at the campuses to provide training and maintenance of ICT facilities. This will afford easy access to ICT services for learner nurses and nurse educators. An office help desk, with a speed dial number, should be established to provide available support regarding the ICT challenges faced by both the learner nurses and nurse educators at the LCN.
- A designated computer room, with broadband Internet access, should be established for regular access by the students.
- All classrooms should be installed with necessary ICT facilities for easy access during teaching and learning.
- The curriculum developers are urged to take a look at the changes required for the inclusion of ICT in the curriculum, so that the college can produce professional nurses who can operate ICT facilities when providing care to the patients.
- College management should invest in developing policies for the use of smartcell phones in the classroom and in the clinical area to support learning.
- Disciplinary procedures should be put in place in terms of such policies to ensure compliance.
- Learner nurses should be formally trained on how to use ICT facilities for learning purposes. The college could achieve this through a partnership with a personal computer training college.
- Learner nurses should be encouraged to use ICT facilities by nurse educators through giving them tasks that incorporate the use of ICT facilities.
- The utilisation of ICT platforms by learner nurses should be monitored, which will result in the provision of quality care to patients.

## 7. Limitations of the study

Framework: The sixth dimension of the el-CDDO framework was not incorporated in the study findings. There is a limited literature regarding the use and application of the el-CDDO framework across the nation profession. Study setting: The study was limited to only two campuses of the Limpopo College of Nursing (LCN) in the Limpopo Province in South Africa, therefore, the current study findings cannot be generalised to other colleges and other provinces in South Africa. Methodology: The richness of the data provided the authors with an understanding of the perceptions of learner nurses and nurse educators regarding the use of ICT facilities in teaching and learning. Data were collected only from undergraduate 4th-level learner nurses, therefore, the perceptions of other levels of study and postgraduate learner nurses regarding ICT are not known. Future studies may be conducted to cover nursing colleges within the province.

## Supporting information

S1 File Sample of interview transcripts data. (ZIP)
