# Supplementary material for: Applying an E-Learning framework to explore learner nurses’ and nurse educators’ perceptions about technology platforms in nursing
Source: PLoS One. 2025 Mar 18;20(3):e0312681. doi: 10.1371/journal.pone.0312681 (PMC11918404; doi:10.1371/journal.pone.0312681)
Supplement: S1 File — (ZIP) [file pone.0312681.s001.zip › sevenga group 1 word document.docx]

**Good evening**

**How are you? My name is Mr Ravele TA, I am a Masters student from the University of Limpopo. Currently on my final year of study it is required for me before I graduate that I conduct this research so that is why I am here to make sure that I meet my objectives. So I have given you the consent form which stipulates that you are giving consent to participate in this study I just want to clarify that if you feel that you need to withdraw from the study you can do that at any time, so nobody is forced to participate in this study. We will be under record with your permission the reason being that I can’t capture everything when we talking so this will help me to go through it when I will be analysing the data. However, nobody is going to get access to these records except people who are involved in the research like my supervisor. That is the only person who is going to get access to this recording, and for security purpose we are not going to use names. I am going to name you as participant 1, participant 2, participant 3, participant 4, participant 5 and participant 6 so we are not going to use any names, so you can feel free to participate. I am conducting a study for the college on the three campuses regarding ICT so my topic is “learner nurses’ and nurse educators’ perception regarding the use of ICT in teaching and learning”. So what are your perception regarding information communication and technology in teaching and learning here in Limpopo College of Nursing?**

**Participant 5:** in the view of this ICT thing in our college for me I feel like we still lacking that kind of can I say way of learning? Because when you check the college I don’t know if I should say poor or something or mismanagement of money to give us good facilities on ICT thing. When you check our library we don’t really use our library per se because we find it not interesting. Even laboratories we don’t even have laboratories the only thing we have there is the doors it’s not even sufficient for our learning. That one with our college ICT thing is not really sufficient for us that we can get enough knowledge on our study.

**Participant 2:** with me my perception about ICT in this college I would say we lack the ICT because with communication between the students and the lecturers it doesn’t involve technologies and again with the library sometimes, some days they don’t even have WIFI or the network when we want to go through the internet and the other thing that I can say with the ICT with this college is doing is that the lecturers they use the projectors during the learning that’s all I can say about the technology that is used here in the college.

**Participant 3:** the way I see or the view this is that we do have facilities we just lack people who can who are well equipped who can help us on how to use these ICT. The reason I am saying this is that we as the college we are in association with University of Limpopo which in my view I think is well equipped in terms of ICT so if we have association with the structure that is advanced in ICT I think that if we had people who are well equipped we can also, they were going to plan our courses in a way that it can make use of this technology because we have a library which is in good standard. I mean you can access internet, computers anytime. The way I see is that our course has not structured by the people who are planning our courses, they didn’t plan it the way that we can see the **shoft** the things of technology as being useful for our course and also I would give an example the computer lab that we have, we have a computer lab that the way our course is we do not have things we do not see it of value that we can go to the computer lab and use spare time trying to acquire information so the way it is I would ask for the course or the people who are planning our course o use strategies so we can see our books be enough for us to acquire information and to explore information through technology and to make use of all this other things that can help us. That’s why we feel comfortable when we are using our books and do not want to go and explore other things.

**Ok, so when you talk about people who will assist in operating who are we looking at?**

The people who are I would say I’m looking at people who will assist us in operating I am referring to people whom, ok let me give an example of the university in the university you will find department called the IT department you will find that those people who work in that department they are responsible to make sure that we still have are aware of the technology things in the company we are aware of communication most of those things, but with our college there is no such department here we say this is our IT department so I believe if we have such department we were not going to be lacking on this information in this technology so those are the people and if we don’t have those people we can say that these are the IT guys who help us with those kind of things we are still going to be lacking.

**Participant 5:** To add onto participant 3: If we check the personnel’s at our schools we are having lecturers who are old fashioned their skills are not well equipped to the new technology things it’s very hard for them to fit in the new technology because if you say or talk about the technology that they use during the lessons they are just copying something from the book as it is so there is no you can see that this person just repeating one thing each and every year to different students there is no advanced in those things even the personnel’s the lecturers they are not well equipped with the new Way of doing things. If we can start with them at least with them we can have a very best way to use this IT thing to our own benefit or to the student benefits.

**Participant 1**: with my view concerning this matter is that on the advantage side is that it was going to be good or it was going to equip us with regard to knowledge on nursing side if we were having internet connections for us to go an extra mile in trying to search certain skills or maybe how to do certain things the problem is that like participant 2 has said sometimes the library there are no connections or WIFI or maybe if we are trying to connect like three the coverage is not that god is very, very slow but if they were advanced I’m quiet sure I believe that we were going to be equipped with nursing skills. The disadvantage side is that we don’t have those things and the people who are suppose to teach us are not there as participant 5 has said. Even the lecturers they don’t even have skills on how to operate these new technology things we even tried to assist them, trying to connect those laptops, or those projectors for them to try to teach us so that is what I can say so far.

**So I hear you participant 1 and participants 5 talking about skills of the lecturers, the lecturers are not being equipped, am I getting you right?** Yes

**Then how do you think your learning is being affected?**

Ok can I say this? Yes I want to answer it like this. We cannot be like self centred to can focusing only on our college only remember we are dealing with lives. If we dealing with life people at Stellenbosch are also dealing with life, people from University of Limpopo are also dealing with life we are also dealing with life, so why must we be different them, you understand. if those people we are on the same track I mean the same focus which is life we have to have same knowledge from this ICT thing. It means now we are really affected negatively so as somebody from Stellenbosch will be like having too much knowledge because they will be above everything, they are above us actually through the scope, the practice now we cannot talk of all the technology they are moving with time. We stuck we really stuck.

**You want to say something P1?**

Yes I want to say something. What I was saying is that the lecturers they only focus on is the content and the books. They only copy things from the books and they paste it on the laptop so that they can display through projector and teach us so the focus there is only on the content and forgetting to know how to operate on the technologies part so it’s affecting us because we are more equipped with regard to the content of the book and not focusing not knowing on how to facilitate things so in this case what will happen if there are new machines at the clinical side and we won’t be able to operate them because we only know the theory part of doing certain procedures but not with the machines but if ever they were focusing on the content but as well as the technology part we were going to be equipped both sides if ever there are new updates we will be able to feed in so it’s really affecting us on that.

**P3:** Basically I think the way, I’ll go back to the point that I indicated earlier on to say that at some point I do blame the people who are responsible to plan for our course or our let me say our course. I’m saying this because the way it is in this college it’s like you are being put in a box to say to you that this is what you must focus on and this is happening because people who are facilitating whom we call our tutors are people who they are not exposed to technology they are not exposed to new information so they want to translate this skills that they have to us the way they are. So I don’t want to use the word that is because they are too old to use this kind of skills but if we check we are not like college like our participant 5 indicated that we are not only people of this college only but when we are done here we have to go outside and we have to compete with other people. So it think it has to start with the people on top like how they do it in the university they just do not take anybody because they just don’t take anybody because you know the content and that you going to be a lecture but they check the qualifications that you have and they check I believe they also check the skills maybe as an individual you have. So also we can, I mean that should be the practice with our college so that we don’t just because this person has been in the practice for 20 years and maybe has a certificate in nursing education they should go or qualify or they free to go lecture there and go practice we would forever be lacking in terms of technology.

**Participant 6, you wanted to say something**?:

**P6:** I wanted to say like ICT here in Limpopo college of nursing is so poor because if we can look at for example our results are only published in the notice board, for example if you are to write a supplementary examination and you have already gone home, you have to come back and you won’t have enough time to prepare for your exams so it lead to students not to perform.

**Participant 4, do you want to say something?** Just to add on what participant 6 said. I think in our college the ICT we don’t actually utilise, it we do have but we don’t utilise it. Our method of learning is fixed we only focusing on the prescribed text books. We don’t find much interest to go and learn further or investigate further because we have a fixed text book that they use to set for our tests, so we are only focusing on that. So the other thing is that again they do not like I can say the lecturers or management they do not provide the opportunities for us to utilise them as she said is if we were provided with an opportunity like we have been provided with computer labs we are suppose to have our own accounts where in which we can view our results at the end of the year so we don’t utilise that opportunity we only come to the notice board to check our results. And the other things is that even when we write assignments we have to go and print out and submit them as copies. We don’t even use the methods and the other once and we have to do everything hard copy so we don’t actually use the technology in our college.

**Participant 3:** I think I’ll be focusing on the people who are busy structuring the course for us because I mean the way they are doing it. The college itself doesn’t even have a website, so if a college or higher learning institution doesn’t have a website itself that shows that the people who are planning they don’t want to adjust the college to the times that we are leaving in because every time you want to get information maybe from head office or central office you have to pay, catch taxis to get there but if maybe there was a website things were going to be displayed on the website messages were going to be communicated on the website. I mean we are leaving in the world where you have a smart phone you do a lot of things you don’t have to be going up and down, you can do your transactions actually you can do most of the things you normally do on a daily basis. But the college the way its structured people don’t want to move with time they are still stuck maybe in the older time they don’t want to move with time. They do not have websites, they do not even have email, and they do not even have fax. When you need to fax they tell you that you need to fax to head office and somebody from central office will have to maybe distribute various campuses. So that’s why we find that technology is not effective or it’s not being utilised in our college. I still stand that we do have facilities that we can use to facilitate this kind but we do not have human resources that is well equipped to can make sure that the technology is being utilised.

**Maybe if you can clarify a bit for me here participant 3 when you talk about facilities, what facilities are available in this college, the ICT facilities you guys have in this college?**

I heard some participants talking about the WIFI which is at times; they say at times you find that the WIFI has no coverage. Those are some things when you go to other universities you find that they those that the coverage is slow. So we are having one of the best universities I mean the internet there I mean one of the best libraries sorry for that. The internet is there, the personnel that are working in the library are the same personnel than one day when you go to University of Limpopo you will find them working in that library so those people they are well equipped. We do have a laboratory there and there are computers which are still new so those are some of the facilities that I am talking about that if we can have people who are well trained to can help us with those things I mean we can move with that I mean with time.

**Participant 2:** I want to add on what participant 3 is saying about the website we know at these times learners should apply on line but with this college there is no online applications you have to fill the forms and ja there is no online applications and the ICT in this college is very low. And again with what participant 3 is saying there are many computers there, yes they are there but I don’t know when they are being utilised, ja.

**So when you say participant 2 you won’t know when they are going to be utilised what do you mean by that?**

I mean if the ICT was advanced in this college they wouldn’t be there for just being there. We will be as students we will be utilising them for our benefits getting certain information online and doing all those things, researches we will be conducting our researches there typing our researches and they are there I don’t know if they are lack of some things I don’t know the ICT in this college is low. To add on what they are saying we came here 2016 we found those computers there, we found those computers there and we don’t even know if they are working or not. They are just there maybe they are toys. So we need those people to help us to see if those things work or not.

**Participant 6:** I wanted to add on this thing of manual registration its time consuming because sometimes we have to fill the forms and we fill the forms in our own time and sometimes we go to class late and they chase us out and we miss out other things because the lecturers will be busy teaching others and then if you are late and you won’t be get notes.

**So what role does the ICT play in your studies but before we go to your role I heard participant 1 talking about not sufficient in learning, not interesting what did you mean by that when you said not interesting and not sufficient for learning?**

P1: I can put it like this I’ve seen somewhere I think I was using my phone and when I went to this app they were doing suturing, I think they were suturing episiotomy if not, they were doing C-section it was just a computer and there was some sort of a doll there manual doll you can see this thing is more like living. They were doing a C-section showing that this is the procedure and this is this and that. So for us here we you just go to class they just tell you C-section open page what-what, this is how it is done. Why can’t we just see it? At least if we can see of how it is done not like lecturer saying ok we going to demo and they are using Dr. Ngubane who has been there for ages. Can’t they change Dr. Ngubane and put computer that you can see and then the process then the lecture saying this is how we suture it’s not sufficient for us we need something new, we are new we got new minds, we need something new we are new that’s what I mean about not being sufficient.

**What role does ICT play in your teaching and learning process?**

**P1:** I think it plays an important role in terms of time we will learn things very fast unlike learning the manual things so if we had to see them online or maybe get information on our own some of them were going to be easy and we were going to learn a lot of things in a very short space of time. That’s what I can say.

**Participant 3:** I also think that it makes things a lot easier I mean like when you have told that tomorrow for example we will be watching a DVD on how to suture an episiotomy you can be interested in watching such things unlike tomorrow you will be demonstrated on how to suture a wound so you get used to things that you will be seeing because we are living in a digital world we want to see a lot of things in being done in form of digital but when you do it in an ancient style it makes it boring and it doesn’t stimulate that thing in you. So I think it plays a very important role in making learning very interesting. I cannot go outside to canvassing to say to people go to Limpopo College of Nursing there I mean people will tell you that we are there to get the diploma and we go there is no life in this college so you see people are only committed on the books they don’t want it because of the way things are done at the college.

**P5:** I want to add on that one we get to level with other institutions like I indicated before that we are dealing with life, life is life Stellenbosch people are dealing with life, get to level with other people not us being here and someone from Wits is up there. Life is life we are dealing with life so we have to get to the same level like everyone with the same knowledge and skills.

**Participant 4: you what do you say about the role ICT is play in teaching and learning?** I can say it makes things easier for us and the other thing is that we don’t have to take time in doing the manual applications travelling from a longer distance in coming here we just simply apply online if I want to view my results while I’m at home I just log in and check them other than to tell someone to come here and check the notice board so that way I think if we can implement it here it will make things simpler for us.

**P2 do you want to say something?** about the advantage of having ICT, ICT it makes life easier it makes us gain more information like even if I’m in my room like I would just enter a certain page, search information, study everywhere I am and I can’t be carrying books if I’m going to visit somewhere and all those things. So if ICT ill access information wherever I am.

**I heard you guys talking more about projector, you talked about one method of teaching being utilised. Why do you think that happens, what causes that to happen?**

**Participant 5:** yes, what causes that to happen is that like we said with the higher body with the higher body they are just failing to structure their things, they take somebody who qualify to be a professional nurse 25 years back to come and teach someone who is 18 years’ old who got the new technology. The time, the time they differ and those people are not interested in advancing themselves to acquire in new technology. They are still having the old fashion of doing things they are just using the projectors just to copy everything from the books and the worst part of it is that we are also having the textbooks we are seeing what is being projected there so what’s the use of them projecting things that we can see.

**Participant 3:** the other thing that is making us have maybe one method of learning is because some I mean our course has been fixed I mean they will tell you that we will learn by presentation they will tell you that we will learn by assignment but at the end of the day the assignment that they are talking about you just write for fun, you don’t get grades for those assignments. I mean if maybe those assignments they would say there is 2% from the assignment that will contribute to your year mark. It will give us that thing that when we do assignments we doing with all of our hearts maybe we do quiz maybe they tell us that 10% that they quiz will contribute but the only thing that contribute is tests so even the lecturers themselves though are very young those that are young they don’t find it relevant maybe they can expose such kind of learning method because they are not of value I mean when I’m told write an assignment, I will write an assignment because I know that I won’t contribute to anything I will only study when its test time. That’s why we only having lecture method, actually it’s not only lecture because they are copying the text book so if it’s a lecture you read a point something that is a point there then you try to explain to us but you read from sentence number 1 to sentence number 10 I mean it’s not lecture after all its just reading that is projected to us so I don’t know what we call that kind of method.

**So I hear of all of which you guys are saying then what do you think should be done regarding this issue of ICT in Limpopo College of Nursing?**

**Participant 1:** what best is to employ ICT people to come and teach us or teach the students with regard on how to operate those laptops so that even if we had to do research you don’t focus on one person that has the skill but everybody can be able to access or be able to conduct the technology themselves at his own time so it was going to be very much beneficial to those students if we employ IT people.

**Participant 2:** the other thing that can be done I think is that the programme can be changed and should include the assignments, the quizzes in the programmes so that the students can be able to can be forced to access internet and do quizzes on the internet.

**Participant 3:**  you remember that, let me give this example. Patrice Motsepe about Mamelodi Sundowns, not Patrice Motsepe but the lady that bought it before Patrice Motsepe what she did was to first was to take out everybody starting from manager going down. That’s why we have Sundowns that is solid. I don’t see the point of keeping maybe all the people that are supposed to be taking or considering retirement and pension that should come even if we say that we are trying to equipping those people it will be a waste of money to equip somebody who after 5 years you won’t be utilising them. The best thing is to eradicate everybody who we think that maybe they don’t fit maybe in the times that we leaving we deal with young people maybe just like yourself we have mam here those are some of the people we are suppose to be having. You find that in our college we may be count maybe Mr. Ravele 1,2,3 out of maybe over 30 staff we do not have more than 5 people we do not have people who we can say are ancient time but not of modern time.

**Participant 5:** the other thing that you need to take into account that copying is not a problem, copying is not a problem the way the other universities are doing things and other colleges I wish they are doing things in the advanced way modern way. If it means eradicating everyone then let it be. We eradicate everyone we bring new blood. And that we let the magogos go, they are tired, truly speaking they are tired. So we need the new blood the new mind, new technology.

**Participant 4:** I think it might be improved by utilising the internet if maybe they can get an account where we can save all the things that are happening at the college including also the application forms not to come here and waste time to do the manual applications to do everything manually, I think it will be much improved and access information in time.

**Participant 6:** I think maybe there can be in-service training where they will train educators also the students about using the computers and also using the computer lab which is not working and maybe they can train people I mean educators and students and get sufficient internet where they can access everything the presentations our assignments.

Are we allowed to argue? **Ok,**

**Participant 5:** participant 6 is saying ok, we cannot educate someone who is old. Remember when you are getting old even your mind is getting tired. It will take the department ages to educate someone who should be getting an early retirement or whatever retirement go sit down. get someone who is new, fresh 1,2,3 days she can catch almost everything. We got to level with everyone. Let them go.

**Participant2:**  I think in a way in which ICT can play a role to improve our college maybe those people who are trained or to become lecturers they should be considering the age, they should not take those that are old they should take those that are young and would be able to use the internet. They should be able to hire for teaching the students in that way the lectures would be hired and they would be able to keep with the internet thing in this college.

**I heard you participant 3 and participant 5 saying that you have ICT which is not utilised so does it mean that it has never been utilised? What do you mean when you say It’s not utilised or?**

**Participant 3:** I’ve been in this college for almost four years now, I’ve never been in the library and the only time I’ve been in the library was do to copies and I have never been to the computer lab to utilise them, the only time I’ve been to the computer lab was when I was taking IMCI documents because they keep them that side. We do have those facilities but I think they are some of them they are not even aware of that you can facilitate, communication information that are not being utilised. So we do have them just that we do not have a course that is structured in a way that will value the use of the library or the use of computer as long as I have a text book I am fine.

**So with what participant 3 is saying, participant 5 you had an argument with participant 6 not so long. Would you still stand by your argument after what participant had said, would you still disagree with what participant 6 said?**

Yes firmly so because when I came to the college the facility is talking about the computer lab. We saw it there first week, first month several months tim3 going. To my mind I thought maybe it’s just a storeroom storing those computers there. So you can see the only people are in here to, why can’t they train them they also saw that it was going to be a waste of money to train those old people so maybe they are waiting for someone who is fresh someone to come and take over the technology thing. I really thought it was a storeroom storing computers.

**Ok guys I have listened to your arguments and also listened to what you think could be done as well and this is taking us to the end of this interview. I am not sure if there is anybody who would want to say anything before we close.**

**Participant 2:** thank you for conducting this study it will make people aware and the other young group that is coming they will be able to find systems changed and they will be able to use more technology things in this college.

**Ok, thank you as well, thank you for the time this brings us to the end of the interview. You are no longer participant 1, you are no longer participant 2, you are no longer participant 3, you are no longer participant 4 you are no longer participant 5 and you are no longer participant 6. So you are going back to your usual names. Thank you**
