# Supplementary material for: Applying an E-Learning framework to explore learner nurses’ and nurse educators’ perceptions about technology platforms in nursing
Source: PLoS One. 2025 Mar 18;20(3):e0312681. doi: 10.1371/journal.pone.0312681 (PMC11918404; doi:10.1371/journal.pone.0312681)
Supplement: S1 File — (ZIP) [file pone.0312681.s001.zip › sevenga group 2 word document.docx]

**Good evening**

**How are you? My name is Mr Ravele TA, I am a student from UL, University of Limpopo. I am doing my Masters so it’s a requirement for me to conduct this research for me to meet the requirements for the course before I graduate I need to have completed this research. Hence I am here to collect data regarding my topic which is perceptions of learner nurses and lectures or nurse educators regarding the use of ICT in teaching and learning. So I am here to find out the perception to explore and describe your perception regarding ICT. So we won’t be using our names for security reasons or privacy reasons. I will be naming you continuing from the previous group so you are going to be participant number 7, you will be participant number 8, participant number 9, participant number 10, participant number 11, participant number 12, participant number 13, participant number 14, participant number 15 and participant number 16. So those are the identifications which will be going to be using today. I have given you the consent form which you give me permission or which will give you permission to participate in this study as its already been stipulated there that no one is forced to participate in this study if you feel like withdrawing while we are in the middle of the conversations you are allowed to withdraw or to do so. So before we start I would like us to put our phones on silence so we won’t be having any disturbance, if we can do that.**

**As I have already indicated that I'm here to conduct a research on perception of learner nurses regarding information and communication technology in teaching and learning in Limpopo College of Nursing. So what are your perception regarding information communication and technology in teaching and learning? Would you like a clarification?**

**P1, P7 I meant to say, what are your perceptions?** I think the technology might have more benefits especially in the practical, I don’t know if I understood the question or the topic.

**No I’m asking what your perceptions are regarding ICT in teaching and learning, so you can continue.**

First of all, I would start with the practical things. So I think that it will be beneficial because you might find that sometimes in the practical places you are alone and the lecturers and the sisters are not there if you are using the technology it might help you to find the information you are looking for. That’s my point of view.

**Participant number 8:** I want to breathe first. I think it would help because in most cases you might find out that some students don’t understand when the lecturer is busy lecturing, so if you have time to go through your internet or maybe podcast on that certain topic it will help you to understand or maybe **attitude** towards your lecturer’s **attitude** towards you, you cannot stand her or you cannot stand him you can just go through your internet, go through that syllabus or that chapter I think it will help in that way and also it can also help you know things that maybe your lecturer didn’t touch or your book you go deeper into those things and also it saves time and also it’s not that much of a burden because with books you have to carry them everywhere with your internet you have your phone with you. It is helpful it should be helpful. That’s my view.

**Ok do you want to say something?** Yes

**Participant number 13:** I agree with participant number 8 because offloads our things. For example, when you have to go to school we have to carry GNS book these books it’s too big, its heavy but if we have a smart phone just to carry to go to classroom it decreases the loads of us having to go with the heavy books and also it will be better if we have a technology whereby we have a prescribed book inside there it will be better because it would be reviewed and have access to the old and have more information than the new.

**Ok participant number 9:** it is also helpful in terms of communication where our lectures to pass information into us like its more info and they can just tell us what they want to tell us via ICT

**Participant 16:** I disagree, how about if a person doesn’t afford data to access those internets it means they won’t be able to get information from those lecturers and so forth.

**Participant number 14:** maybe on that one maybe they can install WIFI and have some password so that we can enter into that website or email.

**Participant number 13:**  I can see that they should provide small laptops like the University of Venda whereby they provide all of their students with tablets they can do that. Or because we are getting stipend they can take maybe small amount R300 per month for 12 months then they can be provided with the tablet and also WIFI.

**Participant number 8:** I still stand that its very convenient though we can talk about data and WIFI it’s still convenient because you can carry it everywhere and internet is everywhere and technology is everywhere than carrying your old books than all these other things I think it would be very helpful. I so disagree with this data because you can buy data and get data where ever you want and you can access information whenever you want as a student nurse.

**Participant number 7:** I think I can disagree with the thing of buying tables because they might be playful in the classroom they won’t be focused but with the books they won’t be having whatsapp messages and everything so I think books its fine rather than phones.

I think the one providing us with tablets it can be helpful because like imagine having to buy a book now and then because they are always releasing new editions and then we have to buy now and then. But, with the phones it will be more cheaper than buying, having to carry those heavy books every time to class. We are adults we won’t use them when it’s not necessary.

**Participant number 15:** I do not think getting tables or whatever to use or whatever technology or whatever to use in class could be a distraction because even now when we having like books we still have our smart phones in class whether the lecture sees us or not we use them in between the session. So I think we learn differently and if you check these days we more used to tables, we more used to phones we are used to using technology and it makes it convenient really imagine if you studying and you have to look for pages in a whole huge book it makes it difficult but when have a smart phone or a book in your phone you can just type in a work and you get what you want.

It saves time and very convenient. And information is not scattered it’s in one place unlike able to search different books.

**Participant number 12:** according to my perception I think using information (ICT) can be convenient because I feel like most of the information that are in the books they are there in the internet before they can even upload they can make a book they upload it on the internet before so it can be just, everyone can get access to it before it can even be on a hard copy.

**Participant number 10:** practically I think it is convenient cause if maybe you don’t know certain procedures you can just go through your phone and see how they do the procedure.

**What do you mean when you say practically?** I mean when you are at the hospital and you want to do certain procedures you can just go through your phone on watch how they do certain procedures.

**P11:** I agree with both sides of in the class and in practicals that it’s convenient and yes it is convenient because we have ever access in everything that we need.

**So what roles does ICT play in your studies in your learning?**

**Participant number 15:** it plays a huge role in our learning for example as she has already mentioned it’s easy to learn skills on the internet you can just quickly to your YouTube they always have skills there on how to do certain skills yes they have that and also when you need a new word or an explanation you have seen in your book it’s easy to just access it. So I think get more and more knowledgeable when we have our technology.

**Participant number 8:** I agree that it plays a huge role mostly in nursing as nursing is practical like she mentioned if you do not know something you can goggle and understand procedures throughout instead of just like looking through your books. You can read your books and still don’t understand but when you go through YouTube, Podcast or whatever videos for that certain procedure you can be competent it could play a huge role in our learning if we consider using technology it could play a huge role in our studies.

**Participant 15:** I also think that technology helps the school like I said we become more and more knowledgeable so now with technology new things are always uploaded we get updated of what’s happening far worldwide so with that you grow and you learn new things.

**Participant number 7:** I don’t know if we are allowed to include like practical things. Like the one, I was once in Polokwane for practical so there was this other condition extra twin pregnancy and it’s not there in our books so I think technology it updates us with new things than the books which is going to be written by someone and everything so technology is the best I can say that.

**Ok I hear you guys talking about how ICT could have a positive impact in your learning then my question now is how do you think that your learning is affected at this current moment with technology with the ICT status?**

**Can you repeat the question? How do you think your learning is affected?**

**Participant number 9:** do you focus on the positive or both sides? Me, I think like it has a positive impact in our learning because like ok we get updated about latest information and we are able to communicate with our lecturers like when we are in practicals they are able to inform us of when they will be coming and which skills they will be focusing on and then another think like we also get to know other health conditions that are affecting other countries we get to know more information like not only focusing on South Africa but also get to know more information like including other countries we get to nursing like health as a whole. Yes like with me when coming to procedures with technology you can watch a procedure over and over again which was like I don’t think it was going to be possible with the lecturer showing you the same thing over and over again because we don’t catch things in the same rate, some catch it fast and some catch it slow so you can go through your internet and go to YouTube watch whatever the lecturer showed you in the class and find that you didn’t get it and you can watch it on your spare time.

**Participant 8:** so with me currently I don’t think it’s effective because it’s not it’s like a lot of contradictions because we, isn’t it that we are modern? We go through the internet and all that and our lecturers are old fashioned some of the things they don’t really agree with us so you might find out you know something from the internet and she knows something from 1990 and she wants us to produce what was happening then and not what we saw maybe on the updated information so there’s a lot of contradictions it confuses us so it’s not really that effective at the moment.

**Can I add something on that: ok P9:** it’s not technology itself that is affective us negatively, I think what needs to change is the mindset of our lecturers. We need to, let them move with technology you will find that they are still holding on to older information, but if we can bring that technology to them or that new information to them and ensure them maybe also them they can go on the same rate.

**Participant number 8:** with me I think, we are checking the effectiveness of ICT? Like currently I feel like it’s not effective because of confusion like it impacts negatively to our lives. We going to check that and write that and the lecturers are not going to credit that so you know it’s not effective. Its negative, to us its impacting negatively and because we are using technology and its costing us to a point where we lose our marks and sometimes our lecturer don’t understand us and there is a lot of confusions and contradictions between us and the lecturers it’s not that effective according to me this technology at the moment in nursing and learning.

**So participant number 8 I hear you saying you using technology, you using ICT, are there ICT facilities in this college?**

Yes I can say we do have library and computers there by the library.

**Participant number 10 do you want to say something?** No.

**I thought you had your hand up.** The thing is they do allow us to have access or information on internet but the problem they don’t want to put it in writing like they want us to write things from the books so the only thing I think that needs to be done is for like make our lecturers understand that things are changing and they should also change and learn and focus on new information rather than holding on like outdated information.

**Participant number 12: how well do you think your learning is affected?** I’m not sure if mine is relevant but in a practical area for example in hospital when we checking the telephone etiquette in t3erms of cellphones and everything it contra indicates with us using a cellphone in front of a patient for example if you want to check a certain procedure how it’s done. If you don’t tell a patient that I’m going to use my cellphone they will think that you’re on whatsapp, we are doing something different that is not in relation to the procedure that we want do so in some cases it’s not really effective or it’s not really working because of the hospital management or their rules contraindicates with the technologies that we want to use at least to acquire more knowledge about certain things so I think that something we can do is maybe look into details the use of this and make a principle or a rule that can allow us to use cellphones or anything that can help us get access to information that we can use at that moment.

**So when you talk about contradiction management, contradiction with the use of ICT are you, what are you referring to?**

What I’m referring to for example we said some certain skills we use in hospitals we watch them over our YouTube or podcast and everything then if you want do that certain skills at that moment and there is a patient in front of you the patient might think that you are taking her pictures, you are doing something different. The rules of the hospitals or about telephone etiquette doesn’t allow us to use a cellphone in front of a patient so it won’t be easy for us to get access to information at that point on how to do a certain skill.

**Participant number 9:** I think that one is easy move away from the patient and come back and do whatever that you wanted to do. Because the only thing that they are not allowed is to use it in front of the patient.

**Participant number 14 how do you think your learning is affected?**  I think it’s affected because someone said about the information our lecturers do have but they don’t go on the internet they just teaching out of those old books. They don’t go through internet and when you raise some information from the internet they don’t allow us they don’t even mark it so that’s our challenge.

**Participant number 13:** this thing in our learning is affecting because if maybe we have technology or a phone or where we have a link of all the books maybe from whatever the first edition until today it’s going to be better so right now we don’t have that link maybe if provided that it was going to be better. You may find that in this school they want 8 marks and it’s half and the current booking we are having its a hard copy and it maybe has 2 points right now then if we have technology that is better with the link with the old books or other version or I don’t know they can be better or with better education. That’s what I can say that right now if we have technology is going to provide with better education.

**So I hear most of you are talking about how your learning is affected as far as theory is related. Does it have, is there any, how is your practical affected as well? I’m saying, remember I asked how do you think your learning affected so I think most of you are talking about theory, so my question is does the same goes practical part because some of you mentioned something about in practical.**

**Participant number 8:** for me participant number 8 I think for practicals if effective ICT we go through a lot because we go through whatever we go through maybe videos, podcast or whatsoever and go practice what we saw on the internet. So if you saw a certain procedure on the internet and you can do it then the technology is effective. So in practical areas is very effective it’s ok and if you not competent enough you can go through your videos, you can go through the internet and understand certain things and you can do them better so it’s very effective compare to theory that’s all I can say.

**Participant number 11:** its not the same effect as the theory part because the I can give an example of the time we were supposed to do the homne sign on the internet the described it in another way and the lecture didn’t want it to be applied in that way then which means it’s the same as them not having the new information of how to do things cause the didn’t know how to do the homen ssign as they are doing it on the internet they wanted their own the one that they know and not from the internet.

**Participant number 8:**  but I think that is like part of theory because we doing it for marks like class.

P11: No but its practical its not theory, you have to do it practically.

P8: Ok its fine let me finish. Isn’t it that we doing it for the patient if you are doing it and its effective and the patient is ok then its effective like its effective because you know how to do it and the patient at the end of the day is well cared of.

P11: So you are no longer sure if you are doing the right thing as the lecturer is teaching you other thing and the internet is teaching you the other.

**So what are you doing in that situation, what happens?**

P11: We do what the lecturer wants for marks. And you can’t argue with them.

P8: So at the end of the day there is a lot of confusion in nursing learning because of ICT. So it’s impacting negatively. Generally, it’s positive but in nursing at this moment is negative, because there is a lot of confusion like you saying practically this is what is happening but theory also. So yes in nursing it doesn’t have a good impact.

**I hear you participant number 8 saying that in nursing it doesn’t have good impact. Is it, what are you referring to when you say in nursing, is it in nursing in general?**

In nursing because we are not sure about the Universities. But when we are exposed in the practical we are like people from different universities people in different institutions we are they are also confused you ask this one she is going to show you what she knows the other one will say that’s not how we do it so you come up with your own from the internet wrong so there is a lot there is a lot of confusion. ICT in nursing is confusing everyone that’s as far as I’m concerned.

**Do guys I’ve listened to you talking about the impact which ICT has and how your learning is affected at the moment. What do you think should be done, what do you think should be done?**

**Participant number 14:** it’s all free WIFI so that we can get access to the information and it will be cheaper for everyone even lecturers can get the information. So it won’t be expensive, so free WIFI.

**So you had your hand up participant number 9:** free WIFI won’t make a change even now we have data we can access the information but our lecturers they need someone who can make them understand that. In-service training.

I was raising my hand for that, participant number 8. We need education, in-service training fir that. Practicals and theory like in the hospitals once in a while someone to show them what maybe they should perform certain skills from the internet. Shows people how to use the internet also all the technology in the hospitals, show everyone on how to use them. To clear the confusion or the theory as well our the lecturer to get to experience that and that can clear everything. I think that’s proper.

**Participant number 9:** and then coming back to the WIFI one they can install free WIFI at the hospitals there they need it and also put computers or anything that can help the health care providers that can access whatever they want to access cause I think it’s one of the reasons that they don’t get it when we come up with things that we saw on the internet. So when we have free WIFI and we have the things to use we can just show them that you can do things like this like you go here and they will be on the same level as us.

**Participant number 11 you had your hand up, what do you think should be done?** In-services. I think in-service will do.

**Participant number 15:** I think in classroom settings we could replace hard copies with soft copies so that everybody could get to new information updated whenever it’s updated instead of having old books and carrying them around that will make it easier. And also the lecturers instead of they have this tendency of holding on to the thing they were using in the past so but now if they get updated as well and to use soft copies instead of hard copies and be taught on how to update them frequently we can move with time.

**Participant number 16:** maybe we should also be allowed to write internet or google when we are writing so that the man can check if the information the information is correct or not to add on the memorandum.

**What do you mean when you say you should be allowed to write google or internet?**

P16: I mean I add answers those that don’t appear on the book and also write my reference so that they can be able to check for themselves so that those answers are correct.

**Participant number 8:** I think also provision they should provide these computers, they should provide these WIFI they should provide all this technology facilities that we need as students in order to learn properly and as if there is provision for those things they will get to learn through in-service and education and how to understand how technology works and its going to benefit all of us in nursing I think.

**So participant number 8 when you’re saying they should provide equipments, what do you mean by that? Because I think somebody saying I don’t remember which participant who said we have ICT facilities.**

P8: But we have limited resources so they can just add more to that, we have few computers in the library that’s the only technology thing we have I think because whenever they are demonstrating procedures there’s this manual we don’t have anything which has to do with technology. Oh yes we have TV. The videos are old. We do have its very few its limited.

On top of it its outdated.

**Participant number 9:** I think it’s going to be helpful if the lecturers make uh, they allow us to make use of the information from the internet. Sometimes it’s useless to go through internet while you know whenever you write something from the internet they are not going to credit it so we stuck on the books 24/7 so if they allow us to write information from the internet we know we go and get information from the internet and put it in the exam.

**Participant number 10 what do you say?** Yeah I agree with them they should just be updated on new information and they should get used to the internet and technologies so that they should just get more information. Yes that’s all I can say.

**Number 12:** I agree our lecturers need in-service training about all these things and they should allow us to go through the internet and if there is something interesting about a certain topic that we’ve learned in class they should allow us as a group to come with it and present so that everyone can see even them can see that this thing happened everywhere, anytime.

**Participant number 7:**  can I disagree with the technology things I we were talking about they should buy, they should buy its expensive guys like WIFI is expensive, buying computers is expensive sticking with the book thing is perfect.

**Participant number 8:** I disagree with that because we not, I understand I said there should be provision of facilities but it’s not only that but they should be people whenever we present something from the internet movies or from TV or from wherever we saw it they should be open to learn and understand how it works that would help not that they should buy. If they can afford to buy then they can buy allow us to buy and use those things. That’s what we want from them.

But you were talking about those WIFI things, each every m? even though you are buying data what is the difference

**Participant number 13:** I disagree agree with participant number 7 you saying that its expensive while I’m comparing the books that we are buying every year and with the WIFI and the tablet you just buy it once. You gonna use it first year, second year, third year, fourth year with your prescribed book inside. In first year how many books do we have to buy, second year? It’s a lot, third its worse and they get lost along the way and torn. So if maybe we can be provided with WIFI, WIFI is better we going to pay 150 if we pay its fine and the tablet we are going to have it maybe for 4 years we don’t have to buy books every year so tablet I think it helps rather than hard copies.

**Participant number 7: you said its expensive, what do you mean when you say its expensive?**

Like when you buy data every month or WIFI every month but with the book you can. Maybe technology is expensive that’s what I’m saying as for a book you just buy one book.

**Participant number 15:** I think you have to be willing to pay in order to get updated or to keep yourself updated. I mean right now obviously you have your phone, you have your whatsapp there you have your social medias and whatever’s you always buying data to keep yourself updated. So now if you want to keep yourself updated you have to pay. Imagine you have to buy a hard copy for biology for first year by the time you get to fifth year or forth year whatever its already outdated they have already written something new so you need to update. You going to buy another book later on if you want to keep studying biology so now same difference you need to get yourself updated and yes.

**Participant number 9:** in terms of WIFI and data being expensive like here level one you are doing BLS and whatever you use those books for maybe 2 years and then level 3 you have to buy another book. So with internet, with software like if you don’t want that thing you just delete it and then install something new that you want to use and then what are we going to do with those books that we used in level 1? They are no longer buying, they don’t buy, they are outdated they need new once.

**Ok I hear what you saying, what you saying regarding what needs to be done then how do you think, what impact would that with all the suggestions that you are saying, what impact is it going to have in your learning? Some of you were saying they need to install WIFI, they need to have you mentioned the issue of installing computers; you also mentioned the issue of soft copies. How is that all going to have an impact in your learning?**

**Participant 8:** I think it’s going to be time saving and its going to be like very time convenient like for example at the moment we have GNS files that we have to submit. Imagine just uploading them online then submitting them online while you learning that studying GNS for exams so it’s going to be time saving and its going to be very convenient for students and its going to give them time to have social life and also manage their academics and everything. Because now we are running out of time because you have to study for exams and you have to compile the hard copy which could be done via software and upload it and mam get it on the internet or software something like that.

**Participant number 13:** yes, I can say it can be convenient like us these days we spend a lot of out time using our phones it’s going to be easy for us to go through our phones instead we spend lots of our time doing whatsapp or whatever its going to be easy for us maybe doing page 14 or whatever its going to help us when we are studying. On my side I become bored when I don’t get to the page that I’m looking for while I want to study something, so it’s going to be easy for me while I’m studying, while I’m sleeping here I can go through page number 1 so its going to be convenient if we have technology.

**Number 15:** I believe it can have both negative and positive impact on our learning because sometimes imagine you are studying and you get notifications from facebook and instagram and all the other things so it can be distracting but at the same time its convenient as they’ve said you just search what you want and you get it fast, without having to page through the book and get tired having to look for something. And also books online are cheaper I’ve noted instead of buying a book for R1000 hard copy you get it for R40 online. So it’s much convenient.

Participant number 15 I hear you saying it can destruct you when you are studying instagram messages, notifications so I think it’s better because you are going to download the book so its not like you are always going to open mobile data.

You can just mute the notifications if you want to study.

**Participant number 8:** I want to support the muting of notifications I feel like everything comes with self discipline you know what you are doing so this one won’t be on ICT its going to be on you just discipline and do whatever you are there for instead of wasting time and continue. I think this will make nursing easy to learn, everyone would enjoy nursing and everyone would be interested in learning nursing even younger people would enjoy nursing because old people whatever they are doing is old fashioned. Imagine you are looking for books which were used in 2000 and that’s not challenging we want something challenging.

**Participant number 14 what are you saying ?** under technology I think if we have technology in our school it will be better even the lecturers will communjicate with us easy for example they can communicate with us via email, they don’t even know how to use email. Some of them they don’t even know how to use whatsapp so itwill be better because you find out that if they want to pass on a message they call the class rep, what if the class rep forgot so thats our challenge. And even results we just go there manually to check. We can have a website and go in there and check our results.

**Number 15 what do you say? I’m coming back to you number 9:** as they’ve mentioned that it will be more easy and our learning will be more engineered and again it’s easy to memorise work if you saw it on the internet more than on the book so maybe we can get distinctions. We don’t even have to carry books around we can study while travelling.

Even when you go home, even when you using an elevator in mall of the north you can study, and toilet it’s very convenient. People like reading when they there you can and that’s where we make our great decisions anyway.

**Number 9 you had your hand up, you wanted to say something, you forgot?** Oh yes I wanted to support what participant number 14, like they are still using the notice board to update us so its going to be easy like you just go into the internet and if they have a link we want to check our results we can just click the link and check our results rather than going back to check the results sometimes you find that its during recess and you are at home and having to send someone to go to school to check whether you passed or not.

Technology would be great for Limpopo College of Nursing because I think University they have black boards they are using and other universities have some but they get everything they uploading. But Limpopo College of Nursing it’s so outdated, its hard copies and there is nothing new and verbal.

**Participant number 8 what do you mean when you say blackboard, notice blackboard?** I don’t really understand what is blackboard but I think in UL, University of Limpopo they use something like that where you get your results there, where they upload your things there. When they want to send you assignment they upload them there I think universities use that but they have different names they use so yes. But Limpopo College of Nursing we don’t have something like that it’s like hard copy, file you have to submit if they have an assignment for you they have to tell you have to submit it there and they have to tell you and if you don’t have to submit online is something like that and they don’t allow lying information so I think it will help us a lot.

**Alright guys I have listened to your arguments and your perceptions regarding the use of ICT in Limpopo College of Nursing and I think we have come to the end of this interview. I would like to offer this opportunity if there is anybody who would like to say something before we close?** No

**Before we close this session I would like to denounce you, you are no longer participant number 7 you going back to your name, you are no longer participant number 8, you are no longer participant number 9, you are no longer participant number 10, you are no longer participant number 11, you are no longer participant number 12, you are no longer participant number 13, you are no longer participant number 14, you are no longer participant number 15 and you are no longer participant number 16. You are all going to your names. Alright thank you very much for this opportunity, I appreciate it so much.**
