# Supplementary material for: Applying an E-Learning framework to explore learner nurses’ and nurse educators’ perceptions about technology platforms in nursing
Source: PLoS One. 2025 Mar 18;20(3):e0312681. doi: 10.1371/journal.pone.0312681 (PMC11918404; doi:10.1371/journal.pone.0312681)
Supplement: S1 File — (ZIP) [file pone.0312681.s001.zip › SOVENGA LECTURERS.docx]

**Good morning everyone my name is Mr. Ravele TA, I'm a student from University of Limpopo I am currently doing my masters so it is a requirement that I conduct research to qualify for the degree that I am doing which is Masters in Nursing Education, so I’m here today to collate data. My topic is perception of learner nurses and nurse educators regarding ICT facilities in teaching and learning. Before we start I would like us to put our phones on silence so that we won’t have disturbances and I’ve distributed the consent form which also stipulates that you are allowed to withdraw from participation any point should you feel uncomfortable to continue with the participation. So feel free to do should you get to that point. We won’t be using our real names for privacy purpose for now or for this purpose I would be calling you participant. So you are going to be participant 1, participant number 2, then participant number 3, participant number 4, participant number 5, participant number 6, participant number 7 and participant number 8. So I would also reassure you that the information that is going to be collected here no one is going to get access except me and my supervisor.**

**So what are your perceptions regarding information and communication technology in teaching and learning here in Limpopo College of Nursing?**

How do we answer by line or we just.

**Participant number.**

Ok how long are we going to take?

**A maximum of 45 minutes.**

**Participant number 7:** according to me ICT is not taken really seriously at our institution or at the Limpopo College of Nursing. I’m saying this because it's not even included in the orientation of staff. When you come as a new staff you’ll never be orientated to say maybe this is how we would communicate. The only thing that we have is the library and at the library we do not use ICT as such but it’s for books mainly, but I was hoping that as educators we would have a formal orientation on internet or ICT as a whole so that we as lecturers also can use the ICT the communication system to communicate amongst ourselves. We even don’t have email addresses you have to have your own personal email address to address issues of education in the college. Thank you.

**Participant number 4:** I think, we are not using technology adequately like now we don’t have the equipment, we don’t have the laptops and then we have the computers in the offices but unfortunately they have lots of viruses so they are not working properly and I don’t think we are using information technology adequately.

**Participant number 6:** I believe if we need to use this information communication technology we need to have facilities like WIFI in the institution which is not there we do try as lecturers sometimes but we are using our own means like we have our personal WIFI. Sometimes we need to, we feel that you need to show them a video but then you need to take your own, these Telkom things then you use it in the class here in the college we don’t have WIFI which I feel it’s a need.

**Participant number 8 noted:** ICT on its own its quiet relevant to the institution particularly looking at the era that we are in. But unfortunately in our institution it’s not practical yes sometimes we do have the resources but are not in good working conditions. Now this makes the ICT not to be considered, where even in management you will find that somehow it’s discouraged where you find that when you have to email a test to one of your colleagues maybe in Thohoyandou or Giyani management still discourage that. They’ll prefer to send the test as a hard copy so you can see that we are not going anywhere with the ICT because according to me I can just an email and my colleague could receive it and we modify it there and there. So generally ICT is needed but looking at the nature of our institution we are still very far from that but we recommend that. Thank you.

**Participant number 1 you had your hand up:** I wanted to that with lecturers I don’t think we resisted much to the transaction or change moving with times the only disadvantage is that we have to pop out from your own pocket that is the other thing that is making people to be reluctant in using it unlike if the employer would supply us with means, resources, WIFIs and everything else that is needed for us to can use the ICT effectively.

**Participant number 2:**  it would be nice for me working in the office because I would be communicating with my supervisor through an email for example I would write a report then email it to my supervisor but it takes me quite a long time for my report to reach my supervisor because I have to type, go and print it get a hard copy, there I find my colleagues waiting there is a que there and after printing I have to go to my supervisor to give and still that hard copy at times isn’t it that she has to edit it, but now she has to edit there and I must go again on the computer unlike sending her the email she edits it and send it back that would even save more time. So because of lack of resources I’m unable to do that there and there is a lot of time wasted.

**Noted participant number 4:** the other thing when we want to communicate with our students we have to use our own cellphones because we don’t have this information technology here. We have a room full of computers but they are not working so it’s a problem.

**Participant number 4 what do you mean you have a room full of computers that are not working?**

They are computers they are new and we never used them that is the problem maybe because we don’t have a WIFI or what I don’t understand.

**Participant number 5 you had your hand up:** thank you, at Limpopo College of Nursing here in Sovenga campus we don’t use technology if you want to use technology you’ll use your own money or your own resources even in class its difficult because we use hard copies, we group students and technology is not used even if you feel it is necessary to use technology maybe by giving examples you want to use videos to show students videos is difficult because we don’t have resources.

**Participant number 7 you had your hand up:** my perception is still that the college is not serious about technology why because what I wanted to put forth is even the schedule because we are dealing with marks here we are still using a pen to write students final marks. We do not have a software or whatever that can help us to, even excel ordinary excel is not being used in our college or in our institution the college at large the Limpopo College of Nursing so we have to use red pens, blue pens to put in marks for all the students for the whole college. And technology again is changing over time isn’t it. If they were serious the management or whoever is responsible we were supposed to be having workshops on 6 monthly basis because really technology is really revolving as mam has already indicated that it’s a serious thing we need it to have workshops 6 monthly every year, every time whether you are new or whether you are old whether you exist in the system or going for pension you need to heave a knowledge of technology to deal with students and to offer quality education and even our products, our students as they go to the workplace as they get absorbed in the workplace they must be familiar with these things so what if us the lecturers we are not familiar with these things . What will happen to our products? That’s my input, that’s why I’m saying I perceive this college as not being serious about the changing world that we live in today, thank you.

**Noted participant number 3.**

**Participant number 9:** thank you for this opportunity I support the previous speaker that our college is not serious. Actually it’s not serious about the quality of products we are producing as long as we have a quantity so what is the type of learning we are producing the quality is not serious, they are not serious about it. Why am I saying so? For example we are teaching learning who re technology orientated you cannot go to the class and be lecturing the traditional lecturing the students do not concentrate but the moment you say open up your phones and google 1,2,3,4 everybody gets alive. So but then now in a space where we do not have the resources to be able to provide these types of students we are teaching or to **acment** the technology based students and the other thing we do not have the backup system for example we are dealing records of lifetime here should your computer crush everything is lost concerning the students. If it happens that it crush while you haven’t printed remember the printers also most of the time are offline they are not working due to whatever logistics and then you have saved a lot of things even in the memory sticks but if your computer crush, it crush with your memory stick because of virus and then the information is lost and nobody cares. All they need is just the information where you get it nobody cares. we have computers like the other speaker said, we have computers we have the nice lab but it’s not utilised and its wasteful expenditure so like for example our student I personally use my own money and internet to the class when I want to teach the students using visual learning so you find that you have to portray a video to akment what you were teaching. That’s my own money and I’m not being compensated on that and I cannot do that on daily basis it’s not possible. So those kinds of things even if you bring them forward the management doesn’t care or the department or whoever is responsible doesn't care as long as at the end of the day you say 50 students passed and are graduating but what type of quality is it. So at the end of the day you are jus producing learners who just have a theory that there is this and this in nursing a patient but they don’t actually know that thing they have never seen it in the ward, some they have never seen it in the videos or something like that but if we had enough resources for technology certain things they can see them on the videos that this is what we are trying to say even if you don’t come across it in the ward but they have an idea of what is happening especially to keep them away because with the traditional way of learning the students are not learning anything. Thank you

**Participant number 3 you had your hand up:** let me support the previous speakers that the college is not serious about technology. We don’t even have a technician on campus so if you are having problems with computers you have to consult fellow lecturers or maybe sometimes students to come and assist you. Another thing is that we don’t have the anti-virus software where sometimes you really lose information that you are having. So you don’t even really care to find us a quality anti-virus software.

**I hear what you guys are saying regarding, oh participant number 4 you have something to say?**

Yes, I want to mentioned again that especially towards end of the year where we have to write a schedule if you make a mistake so it’s a problem maybe 4 lecturers write their marks before you and if you make a mistake you must go and do a photocopy come back recopy the other lecturers work and do your work. If you make a mistake is a problem its very much frustrating this thing of not having technology.

**Participant number 4 what do you mean schedule?**

We write marks on the schedule maybe let’s say its midwifery, PNS, GNS. PNS will write, GNS will write, midwifery we write but if you make a mistake you must come and write the GNS, PNS and the starter it’s frustrating.

**Participant number 7:** just to add on that it also affect us as lecturers in Afrikaans we even say we have “oor klaps” because you find that even with the textbooks the students are using we strictly want hard copies of textbooks we want to see them. If the students are having them on their gadgets we become doubtful as lecturers. So you see the issue of technology is still a monster even in us. That’s why we are saying the management should they take it serious then the technology and the communication system will also become serious in the campus because I understand if the students has got the book on the gadget and the other one is having it on the hard copy I mean they are equally useful those 2 gadgets but because also us we are not sure of this and we do not even perceive it maybe as something very important then we want them to come carry those books from psychiatry to GNS thank you.

**Participant number 6:** so I wanted to mention the other thing like the issue of technology was up to standard it was a lot of time because even if the students will get their results through the internet but they have to be placed on the notice board so we are still traditional we are not moving. It will save them time they have to come back from home to come and look at the notice board.

**So as you were talking I heard you mentioning participant number 7 that you have a library and I also heard participant number 4 saying that you have a room which is full of computers but not working. I just wanted to find out what are in the current situation here in Sovenga campus what are the ICT facilities which are available the once which you guys have?**

**Participant number 7:** thank you, as I’ve said the library, we have the library it has got computers but there is no technician even in the library, why because of late if you can go to the library and put your USB there everything goes viral it goes to they say it’s a short cut and there is not show. Isn’t it’s we have a USB show even have a show in the very library that we are using so it’s not user friendly it’s there. It has got google it has got computers, we have user words we have got password but the library also doesn’t have a technician but if it has got a technician I don’t know if librarians are also technicians we have got 3 librarians but I don’t know if ever they are also trained in relation to the viruses or operation of computers because of late really we are struggling. We don’t have any information on our USBs because of the library itself.

**Participant number 1: or are you covered?** You asked us what we are using at the moment. At the moment we are using our cellphones or sometimes we have to go to another office to or maybe to access a computer or the internet. Like myself now my computer is not working and then I have to go to my next door maybe if I want to write something from the computer so it’s frustrating really.

**Participant number 9:** ok myself I try to improvise or to I don’t know where to use or yes ill use the word improvise. In class for example remember we do not have a WIFI internet in the college or in the campus. Sometimes when we are busy in class you find that there are some things that we need to google so when you say to student google on your phone they complain about their data of which to me its relevant even though it’s for their own benefit they would complain that they do not have data and I cannot force them to google things at their cost or maybe if they do not have data how are they going to google. So even our textbooks that we are using most of them they are old they are not updated so we need the current information of what is happening at the moment and the only source at the moment is internet so but then if we do not have access to the internet how do we bring in the evidence based knowledge to the class. So we always teach students at some point we fill embarrassed we teach students that this and this is what is happening in the management of a certain condition and the student say now these days they do this so because it’s what in the clinical practice they are doing at the current moment to the evidence based. So we are far because we cannot access information so remember internet brings the whole world to your space instantly so even there are eBooks and whatever but you cannot access them because we do not have internet even in my office I do not have internet I utilise my own internet so for you to provide proper education we need to improvise we use money from your pocket and at the end of the day we want that quality nurse who will be able to function efficiently in the hospitals. But then when they do not practice that way after they have graduated we raise our ears we say but we taught them but what is it that we taught them. We taught them the old knowledge and things have changed but we couldn’t have a space where we can embrace the new literature that is available because we do not have the recourses we don’t have the current books, we do not have access to the digital books because of lack of this technology thing.

**Participant number 2:** she has just reminded me of, I’m trying to support the lack of resources like let me give an example of the discipline that I’m in you find that we are only having 2 laptops and there is this I’m giving examples there is this so called bosberad meeting and then the one that is attending obviously will take one laptop with her. Now the remaining lecturers who are suppose to go to class use the technology will be having one laptop and then that’s why there is problem because one should use what the book, the hard copies or what whilst one is utilising say its level 2 and level 4 and classes are running concurrently. So now she will have to have that laptop and I will have to remain and another challenge is that if the very same laptop she is having is having a problem we are both stuck. So technology wise we are not covered.

**Participant number 5:** thank you, I am participant number 5 and I am still on the point of laptop. In my department we are 5 sharing 1 laptop which is currently crushed because of the viruses because we are using different USBs as some are having viruses and the laptop is not working and then the overhead projector we are sharing one being 2 departments. It’s a problem because we are having same periods at the same time for both disciplines and then as for internet because some of the offices we are having internet in the office which is always not accessible. The internet is only in the office if I want to use internet in the class there is a problem so I don’t see it being useful because it’s only in the office and I was also not trained or in-serviced on how to access some of the information. So it’s only there only to check maybe the news the google or I don’t use it for teaching and learning so I think we need to be trained or in-serviced on how to use that internet.

**Participant number 7 you had your hand up:** I thank you very much for giving me this opportunity you will bare with us researcher your topic is a burning issue its really a burning issue. That’s why we want to say everything with the hope that maybe you’ll come up with some recommendation and we will be saved. What I want to say now is adding to what my colleague has already mentioned. What do we do when we have got this challenge? Sometimes we do trial and error or we just act on hear says, how. There is this thing called free viruses, free anti viruses on google. You know sometimes I’ll spend half a day trying to access or download the free antivirus but because I’m not trained I don’t know how to do it. You find that I’m failing because there is this thing that says free antivirus for 30 days or 6 months or so much so you’ll find me trying to access and access until I give up. Hence we say training is the best to show seriousness.

**Participant number 9:** alright uh researcher you are raising our emotions now. I never thought that this is serious but now I can feel it’s serious because it’s taking us back. As colleagues we are also fighting due to this issue of technology, why. We do not have anti viruses so some of use we’ve got, we’ve installed our own anti viruses in the campus computers. The computers that are allocated to us I have put my own antivirus I don’t take any one’s memory stick in my computer so now colleagues come they want information from my computer and I say no, then they say this is a state computer. Is the state computer serviced and managed or maintained by me as allocated to me? And then another lecturer want to put his or her memory stick in that computer allocated to me which is maintained by me for free virus. Os now when you disagree with that person so there is that commotion we start to fight saying you are trying to own this thing we want the information you see. Sometimes we want to print the information for the person the printer is not working and so this is a very serious problem because if the person put their memory stick all your information is gone and nobody cares the next thing you are going to class and you don’t have the lessons plans, you don’t have the schedules you are just going to start afresh and nobody cares they want all those things and you as the lecturers also you need to go to class but then now where is the information, the information crushed because of someone’s memory stick. The virus is very dangerous so it has caused a lot of.

Allow me Mr researcher.

**Participant number 7:** I wanted to tell you that we are not just saying these things. I even went to incredible connection to buy an antivirus called **Capeski** and then it has got information on the box and it says it lasts a year and you must activate it. I brought the box I tried to install it you know what I failed it expired without working. It even expired it’s still there the box is still there in my office I failed. And then there was somebody who was coming here to install the ant viruses I asked about it and he just said I don’t know maybe it will be overload antivirus. But his own antivirus he was supposed to install also failed he said he is coming back but they never came back. Because I did it that one failed because I thought maybe mine will work but it didn’t work so we are frustrated the department or Limpopo College or whoever is responsible for teaching and learning students is not serious that’s my perception, thank you.

**Participant number 6 you had your hand up:** participant number 6 I’m thinking to say what quality products are we producing as we are training our students because we are not using any ICT. Because when they go to the workplace as I once came across one professional nurse to say at the workplace there are challenged because they can’t use computers sometimes they need reports that are typed but some of their trainings they were never exposed to ICT so they find themselves challenged and as they work with other team they work with physio's other people they find themselves very, not having the skill on the computer so they find themselves challenged. So my question is now how many students that we are training know how to use the computer? That’s my concern because right now they qualify without knowing how to use the computer.

**So with all this which you are saying regarding ICT how is it affecting your teaching?**

**Participant number 9, I’ll come back to you participant number 5:** like we’ve already admitted that it is impacting on the quality of learners we are producing. It sort of like we are teaching learners how to cram and go but there is no integration of the real practice with the theory that they are learning because some of the conditions or procedures you might not find them in the wards but if you embrace technology to see in other worlds wheat is the learner that we will produce will be broad minded because of the lot of things that are not there at the end of the day students assumed from level 1 up level 4 they will just say I assumed and assumed because even the very same books as we’ve said they’ve got old information we don’t have new books but if we have ICT we would download the new books and then disburse the new information so even us as lecturers it’s not easy to dish out the information we want to dish out if we teach a patient about spatula if you can’t project it for a student to see a spatula while in the hospital due to lack of resources the spatula is not there the student will just know the spelling of the spatula but the real spatula the student will not know. Like for example right now we talk about suctioning a patient and tracheal sucti9on there are not suctioning equipment in the hospitals due to lack of resources so you just singing to the students that you suction the patient, open airway then the student just know that there is suctioning but they don’t know and they’ve never seen but if there was technology we could post videos show the students how suctioning is done so it really affects the quality of nurses we are producing and we are very far to reaching the I don’t know if its government or the millennium goals or the G4 whatever it is called. So because we are producing nurses that do not have the quality experience in nursing patients.

**Participant number 5:** thank you, it’s having a negative impact on myself because its time consuming. I take a lot of time preparing for lessons because I have to go office to office borrowing books in order to prepare a lesson. So if we were having technology it was going to be easy because I was going to access the books online. Again on the part of students I feel that the lessons are boring because there’s not much to view. We are just, they are just imagining things so our lessons are nor interesting because of lack of resources. So it’s also having a negative impact on students.

**Participant number 8 you had your hand up:** thank you sir just to answer your question of how does it affect teaching and learning. It affects it negatively because as we have indicated that we are using a traditional method which is only your discussion and lecture method so towards the end of the day you find that students are bored because they have seen teaching methods are used remember our classes starts at 7 o’clock up to 16 hour so 7 o’clock to 10 o’clock lecturer method and group discussion 10:30 to 1o’clock lecture method and group discussion towards the end of the day from 1:30 up until 16:30 then you find that students are in class but psychological you can see that they are very bored because of the same teaching strategy that we are using so with ICT it was going to make a difference so in short it is affecting teaching and learning in Limpopo College of Nursing.

**Participant number 3 you had your hand up:** it brings out a lot of frustration on the side of the lecturers because once you lose information you have to start afresh because we don’t have antivirus so we lose a lot of information so if it’s a lesson then we have to start afresh, type another lesson plan and another thing is that it waste time because I go to class with a mobile data projector because we don’t have the fixed one we have to go there all the time so we have to connect sometime you connect when you want to project whatever its not displaying on the screen so there is nobody to assist you on that. You find that maybe 15 or 20 minutes still trying to fix the computer and data projector. So even on the side of communication with the students, let’s say the students are in the clinical area you want to give them an assignment or maybe whatever you have to personally drive and go and give them that assignment if the technology was well advanced you can still communicate with them while they are in clinical area and give them whatever assignment or whatever thing you want to give to tem.

Sorry to highjack participant number 9. I’m just baking the previous speaker another thing that is frustrating is the information remember we are dealing with a lifetime students records here should I pass on, the records must remain but in these days I doubt, if I pass if I don’t wake up tomorrow they will be able to access the records because you save students records tomorrow they are not there. And I’m the only one who knows about that but if we had a backup system where we type the information, we compile the information and we just send it through to the backup system I don’t know. Other institutions that are advanced after capturing the information it goes to ICT so it is saved there. Should anything happen the laptop get lost or you’re PC or there’s fire there is the backup of information. They can still print and do wherever but in our case should anybody come and say I want information for Ngwatle or so and so I'm telling you, you won’t find that information the virus has chowed it and even the manual records also a problem because we thought its a backup but by the time the records are needed maybe by so and so. Even the students records at the students affairs you find that they are not there. We are just having a problem in general that is really affecting us but if we can have a backup system for ICT whereby we know that after I’ve saved the information and I’ve sent it through to ICT or whatever or whoever the department they will save the information as the backup.

**Participant number 5:**  thank you another thing is that as lecturers we become frustrated and angry as mangers tell us to bring hard copies to them because we are not having technology. We use hard copies we submit they get lost they always request to give them the same copies that we have submitted so it’s frustrating for us and then another thing the students do not get up to that information because of lack of technology. We are using old books so they get old information.

**Ok so with all this which you are saying regarding ICT state in this Sovenga campus, what do you think should be done?**

**Participant number 9:** the answers are obvious the researcher I think we have already covered them but then the problem is who we are talking to. We are talking we are raising concerns but we are always told about budget but the most annoying thing actually is that nursing is not taken seriously from National up until to where we are. If nursing was taken serious in every nursing college especially for the government colleges we would be having ICT so because it snot taken serious anything related to nursing training is abundant. It’s up to you lecturers they expect you as a lecturer to work miracles for the students to be educated to be competent. But then now we’ve been complaining we’ve been saying these issues out and they say see what you can do. If the records are lost see what you can do. Not long our computers were stolen you expected to be in class the following day, see what you can do. You don’t even have a computer in front of you but it’s see what you can do. How do I see what I can do in somebody’s house? I can see what I can do in my own house I know my budgets and everything but if I come to your house and then you tell me see what I can do with what because you must give me something to see with. Thank you.

**Noted participant number 4:** I think maybe we should have a technician who must come to the offices maybe once a month to come and fix whatever is broken and then the other thing I think we should have the workshops where they teach us about the new developments with regard to information technology.

**Participant number 8:** ok sir thank you, trying to answer your question. What can be done in Limpopo College of Nursing particularly Sovenga campus? So I think management should be informed and be made aware about the positive effects of ICT so what we have discussed some of the issues include department they include national and others but still at the college the management should be informed about the positive aspects or the advantages of ICT because some other ICT relevant aspects can be done amongst us but if you find that management has negative attitude towards that as we were talking about emailing the tests some are not comfortable with that because maybe they don’t, they are still traditional. Communicating with the students maybe communicating with the students class rep maybe using my cellphone or whatsapp but sometimes it appear that its unprofessional to the management but if the management can be aware of that and be empowered about that then it will be taken into consideration because these are the aspects that can be done in our level without involving much finances from the provincial or from the national or from others. But if we are acquainted and the management I acquainted with what we are doing at the moment definitely we are doing can introduce more. I think that is something that can be done with Sovenga campus with9out including other sectors from outside. Thank you.

**Participant number 1:** I wanted to support participant number 9 what she said that nursing is not being valued because if it was being valued from the department then they will see a need to make improvements on the education system on the nursing education system that we are currently working under because I am sure that they know what is happening outside. They are fully aware of how other institutions are operating so why are they failing to do it for the nursing colleges. Why are not doing anything to help improve as much as we as lecturers we can say these I can do, these I can manage but if we not getting enough support from above then we are not doing anything because its only what I can manage to do that is going to work and which is not much for the students which is not much on the final product because we not doing any justice on the final product, thank you.

**Participant number 6:** I will like to suggest or recommend that we have an ICT officer the department should see to it have a person who is dealing with ICT services for both lecturers and the students to have WIFI even in the classroom then it will improve on our lesson plans on our facilitation in classrooms. And the rest will come together.

**Participant number 9 you had your hand up:** ok colleague I want to sort of differ a bit with participant 8. Our management is very aware that technology is important to teaching. They are aware but we’ve got a very serious problem in nursing in general. Nursing management or nursing is led by old people that have passed their retirement age. 2 they are reluctant to change if they knew things are coming especially with the younger people that are technology orientated to them it’s an insult it’s like a threat and then they don’t want to hear anything. Once you start they don’t want to listen because they feel you are threatening them on their positions or what I don’t know. Secondly or fourthly another big challenge is our managers they are not teaching that’s why they don’t know what we are experiencing. Traditional method was good then but now with this generation that their concentration span is 10 minutes including myself I can’t sit in a class for 30 minutes you will talk alone, I won’t be listening but what I have seen in my class you allowed, you talk for 10 minutes you engage the students. I say to them open up your google let’s look at 1,2,3 you’ll see the whole class being awake they can even go for 7 periods without students sleeping because of technology but at whose expense. Our management doesn’t care from local I’m not talking about nationally. Nationally it’s something else and provincial but they know exactly what our challenges are. They always talk about budget I don’t know for health what are they budgeting for because education and training of nurses is part of budget but if nursing could be taken seriously like other health professions for example like medicine. The way people with medicines are being taught it’s very different from how our students are being taught is because ours, our managers being nurses they devalue our nursing and that is why it's going down. Lets embrace the people who know ICT the young generation in this profession they know about ICT lets embrace them and come up with the thing that technology is here to stay and there is no way in the next few years we will no longer have textbooks in classes it will be ipads. In most schools they are using ipads no more carrying of this big bag so I don’t know how to convince this big management to say people you are going out and we are the once who are remaining and if we don’t fix these things now we are going to be left in a chaos. Thank you

**Ok participant number 7 noted:** I thank you let me support participant number 6 when she says there is really a need for a technician in the department. Because I don’t want us to be relying on another lecturer, we cannot rely on colleagues to get information that is very unprofessional. I cannot be going to young people but that’s what I’m doing now and it’s stressing me. I use young once to help me with technology and its tiring and its delaying my going to class to teach those students for as long as I am still here old as I am I need a technician to come and help me until I reach my retirement age which will be comfortable with. So I support the one who is saying let’s have a technician full time in the campus so that when I’ve got problems I'm class the technician will come before I go to class I find the technician has already put things out for me mine is to go ahead with lecturing. That’s my view of a technician full time on campus.

**Participant number 8 you have your hand up:** thank you sir, I’m still emphasising the point that management should be empowered or be informed about the significant of technology in the classroom. Like one of the participant my colleague indicated that the use of cellphones in the classroom it makes us to be awake. So now if for example it is in my class and I say let’s open our cellphones google this and that you in the presence of my boss or anyone who is part of management may see it how do you allow cellphones in class because somehow. Because of traditional method that she used or he used previously. As my colleague indicated that the traditional method of facilitating was effective then but now it’s not longer effective so but if management is aware of that and is empowered and is made aware that this another method that can make us to be awake from 7 o’clock up until 16:30 then it will be quiet relevant so but if the management is not empowered that is when we will have some differences. One of my colleagues will use then it will differ according to the supervisor then she or she accept that but when it comes to me then it’s a problem that is when students are behaving inappropriately in the classroom because you as the teacher you allow them to use their cellphones in the classroom. Can you see the incompatibility that would be talking about but if the management is made aware and emphasise that ICT in the classroom can be effective then we will be on the right track. Thank you sir.

**Participant number 4:** we previously mentioned that we have a computer lab that is not working I think maybe if they can open that computer lab and let the students and the lecturers utilise it I think it will be better and then the other thing another recommendation is of the installation of the antivirus in our computers. I think it is very much important antivirus in our computers.

**Participant number 9:** the most crucial thing with the antivirus is they’ve installed antivirus in our PCs but they are only for 7 days so if we have a technician they will be able to update our antivirus. Remember antivirus needs to be updated so if it’s not updated you can have it and you’ll still have the virus in the PC so what we need I support the idea of having a technician even though you know treasury and the budget but that’s a recommendation to a technician a full time technician. They know that they will address the issue of antivirus updates in our computers including those in the lab because our memory sticks will be moving from the lab to our own allocated computers in the offices. So I think this issue of updating we are leaving it somewhere because people just thing we need an antivirus in the PCs because of lately we were told that somebody is coming to install antivirus in our laptops it lasts for 7 days from there you are back to Trojan and it crushes everything.

**Alright I have listened to all that you have been saying and I hope that the report which I will provide will be taken into consideration by so saying I would just wanted to draw us your attention that we have come to the end of this interview and before we disburse I will like to denounce you and saying you are no longer participant number 1, you are no longer participant number 2, you are no longer participant number 3, you are no longer participant number 4, you are no longer participant number 5, you are no longer participant number 6, you are no longer participant number 7, you are no longer participant number 8 and you are no longer participant number 9. You go back to your original names now. Thank you very much for this time and opportunity.**

Before you pause or you close or we disburse should you feel to come back you are welcome for any clarity
