# Supplementary material for: Applying an E-Learning framework to explore learner nurses’ and nurse educators’ perceptions about technology platforms in nursing
Source: PLoS One. 2025 Mar 18;20(3):e0312681. doi: 10.1371/journal.pone.0312681 (PMC11918404; doi:10.1371/journal.pone.0312681)
Supplement: S1 File — (ZIP) [file pone.0312681.s001.zip › TNC LECTURERS.docx]

**Good afternoon, how are you? (All answer, "Fine and yourself?") I am fine. My name is Mr Ravele, T A. I am a student from University of Limpopo. I am currently doing masters and it is required that I conduct research to meet the qualification of this degree which I am doing. So I am currently here to collect data and before we start, I would like us to put our phones on silent so that we won't have disturbances. I have also distributed the consent form which also stipulates that you are allowed to withdraw from your participation if you do feel the need to do so. So please feel free at any point during this interview and then my topic is the perception of learner nurses and nurse educators regarding information and communication technology in teaching and learning in Limpopo College of Nursing. So I would like to find out from you guys, before we even go further, I would like to notify you that we will not be using our names. I am going to name you as Participant number 10. You will be Participant number 11. You will be Participant number 12. You will be Participant number 13, and you will be Participant number 14. You will be Participant number 15. You will be Participant number 16, and you will be Participant number 17. So I also want to reassure you that all the information which is going to be discussed here is not going to be published to any other person except me and my supervisor, the one who is in charge of the research. So what are your perceptions regarding information and communication technology in teaching and learning in Limpopo College of Nursing?**

**PARTICIPANT NUMBER 13:** Okay, I am Participant number 13. My view regarding ICT is it is important in nursing education because due to this industrial revolution everyone is supposed to be able to use this ICT in order to transfer the knowledge or to impart the knowledge or to facilitate learning.

**Okay, Participant number 14, what is your view regarding ICT?**

**PARTICIPANT NUMBER 14:** As Participant number 13 have indicated, it is important but the problem is, its availability because there are no resources in the campus currently so it becomes difficult to utilize it and yes, it impacts a lot in the transference of knowledge and imparting of knowledge and learning to students because you cannot communicate effectively without ICT resources, even if you wish to. So it leaves us to resort into the traditional methods of teaching, traditional methods of communicating most of the time.

**So what do you mean when you say you can't communicate effectively?**

**PARTICIPANT NUMBER 14:** At times you would like to send something to the students as quickly as possible. When the students do not have data to come back to you or sometimes you may not have data to send whatever that you want to send to the students because there is no connectivity at the campus. You cannot do that. You must have your own personal data and the students must also have data wherever they are before they can respond back to you. If they do not have data at that moment or data bundles, they cannot come back to you. So communication becomes impacted and it's not only about students only. When you are teaching, you are teaching with other people who are all on a team where you need to communicate with them but it becomes difficult to communicate with another accept maybe using telephone which at the moment the campus do not even have. You must walk or you must use your cell phone to contact a particular person.

**Okay, I hear you. Participant number 16?**

**PARTICIPANT NUMBER 16:** Thank you very much. As Participant number 16, I can say as the lecturer who is employed under Limpopo College of Nursing, it's very difficult. It's too difficult for a lecturer to enhance education of learners without information technology like what the previous speaker has indicated. It's difficult to even communicate with student. In case if you want to make sure that student get information as quick as possible is very difficult and as lecturer, we even feel demoralised. We feel that we are very much. We are far behind the. I mean we are not, how can I put it. It's like we are left behind as far as technology is concerned because we don’t have information technology in our institution. We are lacking behind. If you look at what is happening globally, when you go to the university, they are using ICT to communicate. You go to other colleges, they are using ICT but with our college we are very far, we are behind and it's really affecting us. Not us only, also affecting the students because our student, we can see they are not even, can I say, I can't say learned but they are not. They are not in par with how other students are looking at our institution it's because of this problem here in ICT. Thank you.

**Okay, Participant number 10?**

**PARTICIPANT NUMBER 10:** Thank you. As Participant number 10, I also think that technology enhanced education is very, very important and I think as lecturers we should be having maybe access to ICT but unfortunately we don’t have because I have read that technology enhance education, promote critical thinking amongst students and it also prepare them for as you see the technology in our healthcare system is more advanced. There are more technological equipment in the hospital and if the students are not prepared when they graduate, they won't be able to cope with the technological advancement that are in their workplaces. So I think we should also be able to access ICT and if we look at the students that we are teaching today, the students, they know technology, they are, these generations they know how to communicate with technology whereas I am there using traditional teaching method which becomes boring to them. So I think this, yes, learning with technology will also be helpful to us as lecturers and as students. So if we can maybe get it, it will be helpful. Thank you.

**Okay, Participant number 17, what is your perception regarding ICT and teaching and learning here in this college?**

**PARTICIPANT NUMBER 17:** My perception, I am Participant number 17. My perception towards ICT in our college is that we need it. It's a dire need for us but we don’t have access to it. It's a dire need in the sense that if we want to show students what we are teaching them, like patients who are having wounds or who are being suctioned, we cannot, but if we have ICT, it was going to be easy and enhances their learning because we were going to go to YouTube and check and show them, but we don’t have access to technology. My perception on ICT is that it will, if it is there, it will enhance learning to our students and it can also help lecturers to part information with students with ease, it will make things easy for educators and for also students. And it is boring in the sense that we don’t have ICT and our learners when they complete their studies, they are not coping with those. When they went to conserve maybe in urban areas where they have access to ICT. It's like as if they were not taught because we are not having access to technology hence it is important and my perceptions are that if we were having access to technology, we were going to teach better and students were going to learn better. Thank you.

**Participant number 18?**

**PARTICIPANT NUMBER 18:** 18, thank you, I am Participant number 18. Realising that we are in the fourth industrial revolution. It's obvious that all the institutions need to have ICT so that the dissemination of information must be easier to almost all. Like we are in the rural areas, we need that so that we can be in par with those who are in the urban areas, hence it's easier globally but practically it needs to also be disseminated to us here in the rural areas so that we can also adjust to what is happening in the urban areas. Hence, it's important that all the institution including our institution as lecturers, we also need that. Thank you.

**Participant number 15?**

**PARTICIPANT NUMBER 15:** Okay, as Participant number 15, I am also having the concern with the thing of not having this ICT in our college because sometimes when you are teaching, you can teach in such a way where you will be struggling. On the other way maybe you will be trying to show the students what is it that you are trying to explain but you are failing. Sometimes you can see it was better maybe if you were having something like a video so that they can see it clearly. So I can say, that it's difficult. It's so boring if we don’t have this thing, really. Thank you.

**Okay, Participant number 12?**

**PARTICIPANT NUMBER 12:** As Participant number 12, I think that it's also important to have that IT in our college because everything then we teach, we must, the student must be in the know how is being done, how and when. So we just assume, most of the time we assume that it's supposed to be like this and it's very difficult. You will find that when the student pass, they phone you where they are asking you that what is this means, what is this is working and it's difficult for you as well. So it is very important to have it. Thank you.

**Participant number 11?**

**PARTICIPANT NUMBER 11:** Okay, thank you. I also think it's very much important to have technology in our institution because it will encourage good participation to these kids because if I am there in the class like giving them a lecture, they tend to be bored. They tend to sleep. They tend to take their phone. Isn't it that these kids are like, these learners are like technologically wise? So they are using their phone. While I am standing there giving lecture, there at the back they are using their phones but if I was showing them a video through technology, there were going to be like participating but just because we don’t have technology in our institution it's something else. They tend to play in classes.

**Okay, Participant number 10?**

**PARTICIPANT NUMBER 10:** Okay, I am Participant number 10. So I want to add somebody said something about technology making communication among students and lecturers easy. So if you can look at the way we are working here, we are lagging behind in technology in such a way that we use this old, old noticeboard. If you want to, the timetable we will paste it at the noticeboard, the results everything but the university and maybe some colleges are so advanced in such a way that they use things like student portals and blackboard. They will just log in their portals. Everything will be there. Sometimes even the assignments. So here it's like we are still lagging behind.

Okay, I hear what you are saying, or I hear the perceptions which you guys have regarding ICT. So what I would like to know is that what are the ICT facilities, what are the ICT facilities you have in this campus?

**PARTICIPANT NUMBER 13:** Participant number 13, at present we don’t have any ICT gadget in our institution. We only have our phones that we are using our data as other participant has already indicated. We need Wi-Fi, we need

**PARTICIPANT NUMBER 10:** Okay, I will say yes, we don’t have advanced technological equipment but as for now we have things like computers, data projector is the only thing that we are used to and we only use that to maybe present our lessons and then the student will still be there listening while we present and I don’t think that is promoting maybe active learning. So we still need more. The technological equipment that we have are close to zero like she said.

**So I hear you talking about using a data projector to when giving a lesson so are there any other methods which you are currently using, other ICT method which you are currently using in teaching and learning at present, Participant number?**

**PARTICIPANT NUMBER 18:** Participant number 18. The facilities that we have, we have it's something that we have just improvised realising that we have got our own phones. We communicate with the students just posting some of the things that they need to check in their books, but still it's not that effective. Thank you.

**So how does this have an impact on teaching and learning? I have heard you mentioning the fact that you don’t have ICT in this facility. How is it impacting in your teaching and learning, Participant number 19? Noted, Participant number 14.**

**PARTICIPANT NUMBER 19:** Thank you, Participant number 19, it delays teaching. What was supposed to be taught in a short space of time, you will find that it is taught for a longer period because of not having something to pick up the teaching, like for example, if you are to use the telephone like what the last speaker has just said, you will find students saying that they don’t have data. You will find that there is no network. We have a network problem. You will find that the students will say that they are using a cheaper phone. They cannot. Their phones do not have those facilities that you need to utilise.

So it lengthens the teaching and it also makes the student not to participate actively because if they are using that technology. Some of the technologies are enjoyable meanwhile the students will be learning. Thanks.

**Participant number 14?**

**PARTICIPANT NUMBER 14:** Okay thank you. I am Participant number 14. Actually I wanted to say some of the things that were said by Participant number 19 but I also wanted to say it becomes very much difficult to say students will check something on their own. So it makes students to rely on what the lecturer has prepared and on the textbooks that they are having and yet if the technology was functional in the campus it will be very much easy to say, go and search this and this and this and they will easily go and search for that information on their own instead of relying on the traditional books that we are using. Another thing, it is difficult even for the lecturers themselves because if you need information outside what you are having, the books that we are having, we cannot get that information. You must also have your own data because there is no Wi-Fi within. So you must also have your own data for you to be able to search for information so that whatever that you are going to give to the student who are looking up towards you, is something which is recent and up to date. So it becomes, teaching becomes a heavy burden even to us without technology.

**Participant number 16?**

**PARTICIPANT NUMBER 16:** Thank you very much. It's going to be like a repetition of what my colleagues have already said because as a lecturer, you also feel bored. When you go to class, we are using the same method now and again. You feel bored. It's like we get frustrated or today I am using maybe a lecture. Tomorrow I am using maybe question and answers. The other day you say let them present. Even student end up commenting or can't you use other methods because they can see you are repeating same methods now and again. They also end up not participating like the other participant has just said or they end up not being active. They don’t participate. We are all bored. Both students and lecturer we are bored by the method we are using because of we don’t have ICT in our colleges. Thank you.

**So looking into that, why do you think the situation is as it is? Why, what do you think is making the situations to be as it is at this present moment, Participant number 17, 19 or 20?**

**PARTICIPANT NUMBER 17:** Thank you. I am Participant number 17. I think that which makes this situation worse is because there is no network at our college. Even if we have our phones, sometimes we are unable to use them because there is no network. So if they can make a network for us and also, they can give us a free Wi-Fi, at least we can use the ICT well to deliver or to teach the students. They can also be able to communicate with us if they don’t understand something. They can even go to YouTube by themselves and look at what is it that they should do when doing the procedure or when studying. They can even go and do it in the YouTube but because there is no network that is a big challenge for this college. Thank you.

Participant number 19?

**PARTICIPANT NUMBER 19:** Participant number 19, thank you. There are free Wi-Fi around where one can go and access the information but I don’t think it will be user-friendly because if one will have to go there, one will have to use, if maybe one is given permission one will still have to use own transport, own petrol. I don’t know whether it could be possible to allow classes outside the campus where people can get access of Wi-Fi like what the previous speaker has said. There are many simplified method of understanding many situations like for example, I teach, disaster and if I am using pictures to show what a disaster is, the impact, the effect through technology, I don’t think the students will forget that because some of the situations are real. They will be learning from real things. So access in a form of Wi-Fi, access in the form of the technology itself in such a way that you can reflect it so that all the students view it, could make the situation easier for us but the present situation does not have such facilities. So maybe failure of having those facilities is worsening the teaching and failing us. Thank you.

Okay, Participant number 10?

**PARTICIPANT NUMBER 10:** Okay, I am going to comment on the reason for this situation. I think the department is somehow neglecting us. It should be giving us support like as a college we are higher, an institution which is offering higher education and if you look at other colleges, maybe private, they have all this technology but with us the department is not supporting us. It's like it doesn’t take us seriously. So the lack of resources, I think we should also be trained but recently it's like most of us, we are receiving some computer classes but still, if we don’t have resources, it will be a problem.

**Okay, coming towards the end of our interview, what do you think needs to be done regarding the issue of ICT in this college, what do you think should be done?**

**PARTICIPANT NUMBER 17:** Thank you, Participant number 17. I think what should be done is to first improve the network or maybe they can erect a tower for us to can access the network. Then they can even install the Wi-Fi for us so that we can use this technology anytime we like. Thank you.

Okay, Participant number 19?

**PARTICIPANT NUMBER 19:** Participant number 19, something was started before and it stopped without further implementation. Like it was once said that they would erect something. The English that will enable us to access the internet. I am supporting the network problem that it needs to be solved and it's urgent. The second thing is that we should also have the library for students which have access to those things, which have computers and we need to have somebody, maybe who comes and train us, who comes and assist us who even if that person is not a full-time person but knowing that there is somebody we can phone to assist us technology wise and also to teach us technology wise because there are some of the things which are of late that we don’t know.

So if we are being taught and we are also assisted when there is a problem it will be fine, and it should be urgent because computer related knowledge it's retained if it's being used but if you are not using it, the computer training that we have attended might also fade up because we won't be using it. So it will need that to be done sooner so that we can utilise the little knowledge that we gained about the use of computer. There are many aspects related to the technology and there are new, even new equipment. Some of the equipment they need to be bought so that we can utilise them. Some of them, we might be having them in our phones but not knowing how to utilise them, but we also need to be orientated on that. Thank you.

Participant number 10, oh, Number 16?

**PARTICIPANT NUMBER 16:** Thank you very much. I think what need to be done here is for the college management to take this issue to the Department of Health so that the Department can take it from there and make sure that we have ICT in our college because I think even the Department of Health is very much aware that as a college we need to have ICT because most of the things like maybe guidelines that the government is like coming with and implementing. They still need us to be having ICT. Like today we were bit about an ideal hospital, ideal clinic. We need to be having technology for us to be able to even teach the students on how to use this when they go to, to be serving the community. I think the issue need to be taken higher up to the government so that the government can take this issue serious so that we can end up having this ICT. I think the will consider doing that for us. I don’t know. I thank you.

Participant number 10?

**PARTICIPANT NUMBER 10:** Okay, thank you but most of the things that I wanted to say was said by the previous speaker. So to add on that, I also think that as a learning institution we should also have computer laboratory because now there is no computer laboratory. So it will help the students if there is internet and the computers that they can use to access some study materials and all those things that will help them during their learning. It will also help and enhance their learning and also our teaching strategies. Thank you.

Participant number 19

**PARTICIPANT NUMBER 19:** Thank you. I am Participant number 19. What came to my mind presently is the use, or is not for teaching as such, it's the use of, I don’t know the name of the technology that is used to mark the multiple-choice questions, that within a blink of an eye, I will be done but you will find that the method that we are using, we are still marking one by one, paper by paper following one another but if ever there is those machines that were used long, long ago by other institutions of higher education where successful but we are still not using, not having those machines which can also hasten marking and the early publication of exam or test results. Thank you.

**PARTICIPANT NUMBER 18:** I am Participant number 18. I also want to think that we are not yet late realising that there is this thing of NHI and primary healthcare re-engineering whereby they can also consider the colleges and have ideal college whereby the equipment like we have ideal clinics, we can see them. We are having all the equipment. So I think our management can also highlight that when they are preparing in preparation because we are now also coming for new curriculum that needs the high technology of course and then it means ICT will have to be advanced so that for whatsoever we are going to give to teach to the new students with new curriculum, become easier. Thank you.

Do you want to come again, Participant number 19?

**PARTICIPANT NUMBER 19:** Participant number 19, I wanted to add that benchmarking is also of importance so that they can go to the institutions of higher learning where technology is mostly used and then copy or learn form that institution so that we may at least have as many ICT as possible. Thank you.

**MR RAVELE:**  Okay, Participant number 14, do you want to say something before we close? Are you sorted? Anyone who would like to say something before we warp up? No one?

Okay, so this brings us to the end of the interview. Thank you very much for attending. I hope the recommendations will be taken into consideration. I would like before closing to denounce you and say you are no longer Participant number 10, and you are no longer Participant number 11. You are no longer Participant number 12. You are no longer Participant number 14. You are no longer Participant number 15. You are no longer Participant number 18. You are no longer Participant number 13. You are no longer participant number 16. You are no longer Participant number 19 and you are no longer Participant number 17. You are going back to your original names. Thank you very much.

**SESSION ADJOURNED**
